# The microbial composition of pancreatic ductal adenocarcinoma: a systematic review of 16S rRNA gene sequencing

**DOI:** 10.1097/JS9.0000000000001762

**Published:** 2024-06-14

**Authors:** Nabeel Merali, Tarak Chouari, Casie Sweeney, James Halle-Smith, Maria-Danae Jessel, Bing Wang, James O’ Brien, Satoshi Suyama, José I. Jiménez, Keith J. Roberts, Eirini Velliou, Shivan Sivakumar, Timothy A. Rockall, Ayse Demirkan, Virginia Pedicord, Dongmei Deng, Elisa Giovannetti, Nicola E. Annels, Adam E. Frampton

**Affiliations:** a Minimal Access Therapy Training Unit (MATTU), Royal Surrey Hospital NHS Foundation Trust; b Department of Hepato-Pancreato-Biliary (HPB) Surgery, Royal Surrey County Hospital NHS Foundation Trust; c Section of Oncology, Department of Clinical and Experimental Medicine, Faculty of Health and Medical Science, University of Surrey; d Section of Statistical Multi-Omics, Department of Clinical and Experimental Medicine, Faculty of Health and Medical Science, University of Surrey; e Surrey Institute for People-Centred AI, University of Surrey, Guildford, Surrey; f Cambridge Institute of Therapeutic Immunology and Infectious Disease, University of Cambridge, Cambridge; g Department of Life Sciences, Imperial College London; h Centre for 3D Models of Health and Disease, Division of Surgery and Interventional Science, University College London (UCL), London; i Oncology Department and Institute of Immunology and Immunotherapy, Birmingham Medical School, University of Birmingham; j Hepatobiliary and Pancreatic Surgery Unit, Queen Elizabeth Hospital Birmingham, College of Medical and Dental Sciences, University of Birmingham, Birmingham, UK; k Department of Medical Oncology, VU University Medical Center, Cancer Center Amsterdam; l Department of Preventive Dentistry, Academic Center for Dentistry Amsterdam (ACTA), University of Amsterdam and Vrije Universiteit Amsterdam, Amsterdam, The Netherlands; m Fondazione Pisa per la Scienza, San Giuliano, Italy

**Keywords:** 16sRNA gene, biomarker, cancer therapy, microbiome, pancreatic cancer, pancreatic ductal adenocarcinoma, signature

## Abstract

**Background:**

Pancreatic cancer, specifically pancreatic ductal adenocarcinoma (PDAC), continues to pose a significant clinical and scientific challenge. The most significant finding of recent years is that PDAC tumours harbour their specific microbiome, which differs amongst tumour entities and is distinct from healthy tissue. This review aims to evaluate and summarise all PDAC studies that have used the next-generation technique, 16S rRNA gene amplicon sequencing within each bodily compartment. As well as establishing a causal relationship between PDAC and the microbiome.

**Materials and methods:**

This systematic review was carried out according to the Preferred Reporting Items for Systematic Reviews and Meta-analysis (PRISMA) guidelines. A comprehensive search strategy was designed, and 1727 studies were analysed.

**Results:**

In total, 38 studies were selected for qualitative analysis and summarised significant PDAC bacterial signatures. Despite the growing amount of data provided, we are not able to state a universal 16S rRNA gene microbial signature that can be used for PDAC screening. This is most certainly due to the heterogeneity of the presentation of results, lack of available datasets, and the intrinsic selection bias between studies.

**Conclusion:**

Several key studies have begun to shed light on causality and the influence the microbiome constituents and their produced metabolites could play in tumorigenesis and influencing outcomes. The challenge in this field is to shape the available microbial data into targetable signatures. Making sequenced data readily available is critical, coupled with the coordinated standardisation of data and the need for consensus guidelines in studies investigating the microbiome in PDAC.

## Introduction

Highlights

- Studies have shown that the pancreatic intratumoural microbiome can influence tumourigenesis, chemoresistance, and the immune response to cancer.
- The most significant finding of recent years is that pancreatic ductal adenocarcinoma (PDAC) tumours harbour their specific microbiome, which differs amongst tumour entities and is distinct from healthy tissue.
- Despite the growing amount of data provided, we are not able to state a universal 16S rRNA gene microbial signature that can be used for PDAC screening. This is most certainly due to the heterogeneity of the presentation of results to taxonomic groups coupled with the lack of available datasets and the intrinsic selection bias between studies.
- Majority of the studies did not discuss the removal of contaminants, and this raises a major concern and pitfall of sequencing low microbial biomass samples.
- However, the focus of studies described in this systematic review is most certainly on interrogating the compartmental microbiome in PDAC in terms of understanding its composition and utility as a biomarker for diagnostics, stratification, and prognostication. As the pancreas is an upper gastrointestinal organ that perhaps the bile or duodenal fluid would be more appropriate for looking at the pancreatic cancer microbiome.
- Several key studies have begun to shed light on causality and the influence the microbiome constituents and their produced metabolites could play in tumorigenesis and influencing outcomes.

Pancreatic cancer, specifically pancreatic ductal adenocarcinoma (PDAC), continues to pose a significant clinical and scientific challenge. PDAC is an immunologically ‘cold’ (low immune cell infiltration in the tumour microenvironment) solid tumour with an extremely poor prognosis, and rising incidence and mortality rates^1,2^. Traditionally, poor outcomes in PDAC have been attributed to late clinical presentation and an aggressive disease course. Most patients are ineligible for curative surgery, with chemotherapy and/or radiotherapy being the main treatment options, which are usually not efficient. Overall, there is a high mortality rate associated with late prognosis, resistance to treatment, and metastasis (linked to late diagnosis). Overall, pancreatic cancer is the 7th most common cause of cancer-related mortality worldwide, accounting for 2.6% of all new cases and 4.7% of deaths in 2020^3^. In the UK, 25% of people diagnosed with PDAC are alive at 1 year and 7% at 5 years. https://www.cancerresearchuk.org/health-professional/cancer-statistics/statistics-by-cancer-type/pancreatic-cancer Despite this, a small proportion of patients with PDAC remain disease-free several years postsurgery^4^. The study of these long-term survivors (LTS) may reveal insight into factors that influence survival and may provide novel therapeutic targets to improve outcomes.

The aetiology of PDAC is not well defined. Suggested associations include chronic inflammation, genomics, and increasingly, alteration to the microbiome. Furthermore, dysbiosis, or disturbed balance of microbiota, is a hallmark of many different diseases, including cancer^5^. Suggested mechanisms include the maintenance of a persistent inflammatory state, dysregulation of cellular metabolic processes through the immune cell-microbe-tumour axis, and altering the tumour microenvironment^6^.

Advances in technology have allowed us to gain a more comprehensive knowledge of the human microbiome. Machine learning is increasingly important in microbiology predicting antibiotic resistance and associating human microbiome features with complex host diseases^6,7^. The 16S rRNA gene has been a mainstay of next-generation sequencing-based microbiota analysis. It has the potential to provide taxonomic resolution of bacterial communities at a species and strain level as well as being cost-effective^8,9^. In recent years, a growing body of data generated across the world has demonstrated distinct changes in oral, gut, and intratumoural host bacteria that are thought to influence the host immune response and prognosis of PDAC^5,10–13^. Proxy measures of the pancreatic tumour microbiome such as oral saliva, faeces, and gut samples are commonly used for PDAC. It has now been demonstrated that the pancreas is not a sterile organ, and reflux into the pancreatic duct from the gastrointestinal (GI) tract allows colonisation by gut microbial species^14,15^. Studies have shown that the pancreatic intratumoural microbiome can influence tumourigenesis, chemoresistance, and the immune response to cancer^16^. Several recent studies have started to capture and catalogue the presence of the intratumoral PDAC microbiome^17–19^. Riquelme *et al*.^20^ discovered a distinct ‘microbial signature’ *Seudoxanthomonas-Streptomyces-Saccharopolyspora-Bacillus-Clausii* as predicting long-term survival, possibly due to immune activation caused by greater densities of Cluster of Differentiation 3 and 8 (CD3+ and CD8+) T cells and Granzyme B+ (GzmB) cells. Specific bacterial classes, such as *Gammaproteobacteria*, have been linked to gemcitabine resistance and worse survival following chemotherapy^13^.

The most significant finding of recent years is that PDAC tumours harbour their specific microbiome, which differs amongst tumour entities and is distinct from healthy tissue^17^. Hence, the microbiome has emerged as a novel component of interest for basic and translational science, and a potential prognostic and therapeutic target. This is the first paper of its kind and given the recent acknowledgement of the role of the microbiome in pancreatic cancer with regards to chemotherapy response, the immune microenvironment and survival after surgery, will provide a great resource for the community. This review aims to evaluate and summarise all PDAC studies that have used the next-generation technique, 16S rRNA gene amplicon sequencing within each bodily compartment. Therefore, using a set technique in translational microbiome science to identify specific microbial PDAC targets. This review aims to provide insights into recent progress in this field, clinically contextualise significant findings in studies and identify the hurdles the field must overcome in the future.

## Methods

This systematic review was carried out according to the Preferred Reporting Items for Systematic Reviews and Meta-analysis (PRISMA) guidelines (Supplementary File, PRISMA Checklist, Supplemental Digital Content 1, http://links.lww.com/JS9/C727, Supplemental Digital Content 2, http://links.lww.com/JS9/C728). The systematic review was registered a priori at the International Prospective Register of Systematic Reviews (PROSPERO) database. This study followed the recommendations of the Assessing the Methodological Quality of Systematic Reviews (AMSTAR) guidelines (Supplementary File, AMSTAR Checklist, Supplemental Digital Content 3, http://links.lww.com/JS9/C729)^21^.

The preparation of the research question was based on the PICO strategy, considering diseases of the pancreas and biliary tree (Patient or Problem); microbiota impact (Interest); healthy and benign disease patients (Control group), all outcomes available in the literature were considered in the analysis (Outcome). We followed the Cochrane recommendations for study methodology and the PRISMA 2020 Statement for reporting our results^22^.

A comprehensive search strategy was designed to identify all studies comparing the outcome of 16S rRNA genes sequencing in different bodily compartments in patients with PDAC. The electronic databases EMBASE (Ovid), Medline (Ovid), and Web of Science (WoS): Core Collection were searched until the 16th of March 2024. Studies before the year 2000 were excluded as next-generation sequencing technology was not yet fully established.

Articles were selected from titles and abstracts according to their data relevance and regardless of publication status. Articles with full text inaccessible to authors were not considered. Missing data was clarified by contacting authors directly.

### key-words of search strategy

EMBASE, MEDLINE: pancrea* cancer.ti,ab. OR pancrea* tumour.ti,ab. OR pancre* tumor.ti,ab. OR pancrea* malignancy.ti,ab. OR pancreatic ductal adenocarcinoma.ti,ab. OR pancrea* adenocarcinoma.ti,ab. OR pancrea* carcinoma.ti,ab. AND microbiome.ti,ab. OR microbial.ti,ab. OR dysbiosis.ti,ab. OR microbiota.ti,ab. OR bacterial.ti,ab. OR bacterial signatures.ti,ab.

WoS: ((TI=(pancrea* cancer OR pancrea* tumour OR pancre* tumor OR pancrea* malignancy OR pancreatic ductal adenocarcinoma OR pancrea* adenocarcinoma OR pancrea* carcinoma)) AND TI=(microbiome OR microbial OR dysbiosis OR microbiota OR bacterial OR bacterial signatures)) OR ((AB=(pancrea* cancer OR pancrea* tumour OR pancre* tumor OR pancrea* malignancy OR pancreatic ductal adenocarcinoma OR pancrea* adenocarcinoma OR pancrea* carcinoma)) AND AB=(microbiome OR microbial OR dysbiosis OR microbiota OR bacterial OR bacterial signatures)).

### Inclusion criteria

1. Randomised and nonrandomised controlled trials, prospective and retrospective cohorts, case–control studies, and cross-sectional studies published in the English language.
2. References were also hand-searched to identify further studies relevant to the review.
3. Incorporation of four major microbiome databases; Microbiome BioProject, Genome Sequence Archive (GSA) data repository, Gene Expression Omnibus (GEO) data repository, and the European Nucleotide Archive (ENA) sequencing platform.
4. Study participants were adults (>16 years old) with PDAC, and control subjects who also underwent 16S RNA gene sequencing.
5. The sample collection strategies examined in the study are blood plasma, biofluids (bile, pancreatic and duodenal specimens) via surgery or endoscopic retrograde cholangiopancreatography (ERCP), intraoperative fresh tissue specimens (duodenum and pancreas), archival formalin-fixed paraffin-embedded (FFPE) pancreatic specimens, oral and faecal analysis.
6. Data were collected for authors, date, country of publication, and analysis methods. Factors associated with contamination and microbiota alteration were also recorded. The available 16S rRNA gene sequencing microbiome data were analysed.

### Exclusion criteria

1. Case reports, reviews, abstracts, letters to the editors, research protocols, and congress proceedings.
2. Studies before 2000 and nonhuman PDAC studies and research focusing on preclinical models.
3. Methods not incorporating 16S RNA gene sequencing and tumours not primarily PDAC in origin.

### Data extraction and validation

Following duplicate removal, study selection was performed in three stages following the PRISMA guidelines. Two reviewers independently assessed titles and abstracts for inclusion, each paper being reviewed by two reviewers with conflicts discussed by all three reviewers. The same process was used to identify full-text papers for inclusion. Two of three researchers critically appraised the quality of each study independently, with differences in rating rectified between all three researchers. Data from each study were extracted by two researchers.

### Results

**Figure 1:**
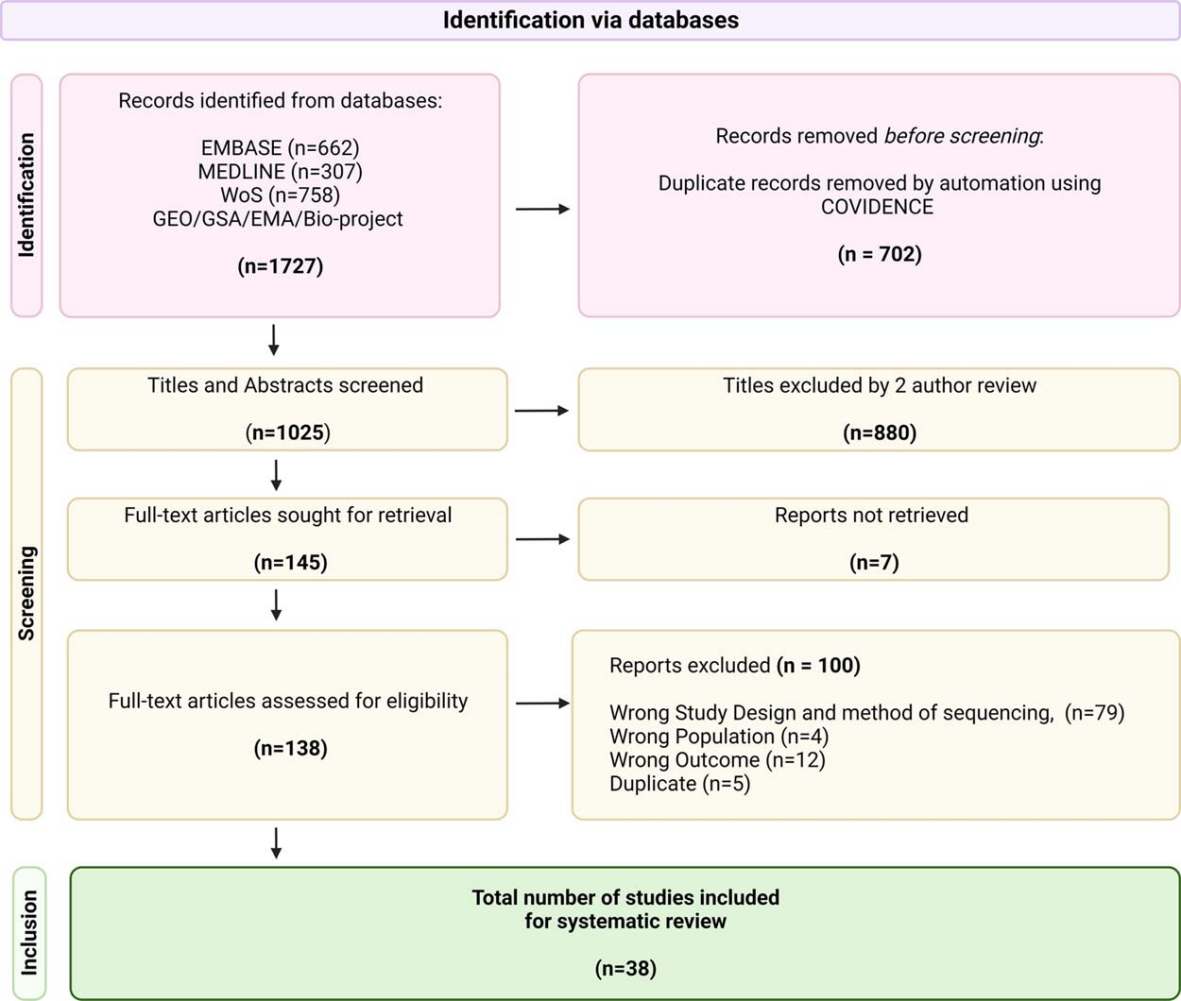
PRISMA flow of the 16S rRNA studies related to PDAC.

The search strategy generated 1727 results. A total of 702 duplicates were removed and 1025 titles and abstracts were screened. One hundred forty-five potentially eligible full-text studies were examined and of those selected, 100 did not meet the inclusion criteria. This included 79 studies that did not use 16S rRNA gene sequencing, four non-PDAC populations, 12 that did not conclude on microbial signatures in PDAC and five further duplicate studies. In total, 38 studies were selected for qualitative analysis and the PRISMA flowchart is shown in Figure 1.

### Quality of the Studies

All the included studies were case–control (*n*=28) or case series (*n*=10). The Newcastle–Ottawa scale^23^ was used for case–control studies, and the Adapted Newcastle–Ottawa scale^24^ for case series. Studies used healthy (or benign) control groups, which were selected from elsewhere in the hospital, or other similarly investigated groups. All of the studies controlled for cancer but did not control for any additional factor such as other exposures known to influence microbiota composition. This method of selection is limited because the only outcome of interest being controlled for is cancer. It was not clarified if the controls without cancer, who were selected from groups undergoing urgent investigation of symptoms, had confounding factors for microbiome 16S rRNA gene analysis, such as an eventual alternative gastrointestinal diagnosis. Some controls were not healthy but had alternative diagnoses, such as pancreatitis, for which the profiling and understanding of the microbiome is not fully established, rendering this group an unreliable control.  The outcome of interest was microbiome composition, therefore there is no loss to follow-up, but there is also not universally accepted the length of the assessment. Confounding factors such as diet, geographical location, and medications including antibiotics are not controlled for in the studies. As this field is novel and evolving, very little knowledge exists regarding alternative causes and triggers for differing microbiome outcomes in this patient group. Therefore, the case series does not examine alternative causes or factors, reducing the total possible score in the modified Newcastle–Ottawa scale.

The studies used a variety of tissue and bodily fluids to examine the microbiota in cancer. This affected case selection; the cohorts were taken from different investigation types, patient groups, and anatomical areas. Discussion of cancer-specific microbiome outcomes for PDAC, and forming a conclusion from the multiple cohorts studied, will be unreliable. There may be unrecognised biases in the results due to uncontrolled case selection. The quality assessment of the selected studies is shown in Tables 1 and 2.

**Table 1:** Newcastle–Ottawa scale for cohort studies

Study (year, country)	Selection (/4)	Comparability (/2)	Outcome (/3)*
Characterization of the salivary microbiome in patients with pancreatic cancer, Torres PJ *et al*.^25^ (2015, USA)	2	0	2
Human oral microbiome and prospective risk for pancreatic cancer: a population-based nested case–control study, Fan XZ *et al*. (2016, USA)	4	2	3
Gut microbial profile analysis by MiSeq sequencing of pancreatic carcinoma patients in China, Ren ZG *et al*.^26^ (2017, China)	3	1	2
Characterization of the duodenal bacterial microbiota in patients with pancreatic head cancer vs. healthy controls, Mei QX *et al*.^27^ (2018, China)	4	0	3
The microbiomes of pancreatic and duodenum tissue overlap and are highly subject specific but differ between pancreatic cancer and noncancer subjects, Del Castillo *et al*.^10^ (2019, USA)	3	1	3
Faecal microbiome signatures of pancreatic cancer patients, Half E *et al*.^28^ (2019, Israel)	3	1	2
Tumour microbiome diversity and composition influence pancreatic cancer outcomes, Riquelme EM *et al*.^20^ (2019, USA)	3	1	2
Characterization of oral microbiome and exploration of potential biomarkers in patients with pancreatic cancer, Sun HY *et al*.^29^ (2020, China)	3	0	2
Oral microbial community composition is associated with pancreatic cancer: A case-control study in Iran, Vogtmann E *et al*.^30^ (2020, USA/Iran)	3	1	3
Oral microbiome and pancreatic cancer, Wei AL *et al*.^31^ (2020, China)	4	2	3
Metataxonomic and metabolic impact of faecal microbiota transplantation from patients with pancreatic cancer into germ-free mice: a pilot study, Genton L *et al*.^32^ (2021, Switzerland)	3	1	3
Composition, diversity, and potential utility of intervention-naïve pancreatic cancer intratumoural microbiome signature profiling via endoscopic ultrasound, Gleeson FC *et al*.^19^ (2022, USA)	2	0	2
Enterococcus spp. have higher fitness for survival, in a pH-dependent manner, in pancreatic juice among duodenal bacterial flora, Itoyama S *et al*. (2021, Japan)	3	0	3
Microbiome markers of pancreatic cancer based on bacteria-derived extracellular vesicles acquired from blood samples: a retrospective propensity score matching analysis, Kim JR *et al*.^33^ (2021, South Korea)	3	2	3
Endoscopic ultrasound (EUS)-guided fine needle biopsy (FNB) formalin fixed paraffin-embedded (FFPE) pancreatic tissue samples are a potential resource for microbiota analysis, Masi AC *et al*.^34^ (2021, UK)	3	1	2
Dysbiotic gut microbiota in pancreatic cancer patients form correlation networks with the oral microbiota and prognostic factors, Matsukawa H *et al*.^35^ (2021, Japan)	3	0	2
Dysbiosis of the duodenal microbiota as a diagnostic marker for pancreaticobiliary cancer, Sugimoto M *et al*.^36^ (2021, Japan)	3	1	2
Biliary tract microbiota similarities in pancreatic ductal adenocarcinoma, Arteta A *et al*. (2022, Colombia)	2	0	3
Integrative analysis of metabolome and gut microbiota in patients with pancreatic ductal adenocarcinoma, Guo X *et al*.^37^ (2022, China)	2	1	3
Changes in intestinal bacteria and imbalances of metabolites induced in the intestines of pancreatic ductal adenocarcinoma patients in a Japanese population: a preliminary result, Hashimoto S *et al*. (2022, Japan)	3	1	2
Intratumor microbiome analysis identifies positive association between megasphaera and survival of chinese patients with pancreatic ductal adenocarcinomas, Huang Y *et al*.^38^ (2022, China)	3	1	2
A faecal microbiota signature with high specificity for pancreatic cancer, Kartal E *et al*.^39^ (2022, Germany/ Spain)	3	2	3
Gallbladder microbiota composition is associated with pancreaticobiliary and gallbladder cancer prognosis, Kirishima M *et al*.^40^ (2022, Japan)	3	0	3
Alterations in the Duodenal Fluid Microbiome of Patients with Pancreatic Cancer, Kohi S *et al*.^11^ (2022, USA)	3	2	3
Characteristics of bile microbiota in cholelithiasis, perihilar cholangiocarcinoma, distal cholangiocarcinoma, and pancreatic cancer, Li Z *et al*.^41^ (2022, China)	2	0	3
Metagenomic identification of microbial signatures predicting pancreatic cancer from a multinational study, Nagata N *et al*.^42^ (2022, Japan)	3	2	3
Bile Microbiome Signatures Associated with Pancreatic Ductal Adenocarcinoma Compared to Benign Disease: A UK Pilot Study, Merali N *et al*.^15^ (2023, UK)	3	2	3
Microbiomic profiles of bile in patients with benign and malignant pancreaticobiliary disease, Shyam K *et al*. (2023, USA)	3	2	3

**Table 2:** Adapted Newcastle–Ottawa scale for case series.

Study (year, country)	Selection (/1)	Ascertainment (/2)	Causality (/2)	Reporting (/1)	Total (/6)^a^
Enrichment of oral microbiota in early cystic precursors to invasive pancreatic cancer, Gaiser RA *et al*.^43^ (2019, Sweden)	1	2	1	1	5
Microbiome patterns in matched bile, duodenal, pancreatic tumour tissue, drainage, and stool samples: association with preoperative stenting and postoperative pancreatic fistula development, Langheinrich M *et al*.^44^ (2020, Germany)	1	2	2	1	6
Comparisons of oral, intestinal, and pancreatic bacterial microbiomes in patients with pancreatic cancer and other gastrointestinal diseases, Chung M *et al*.^45^ (2021, USA)	1	2	2	1	6
Tumour microbiome contributes to an aggressive phenotype in the basal-like subtype of pancreatic cancer, Guo W *et al*.^12^ (2021, China)	1	1	2	1	5
Role of biliary stent and neoadjuvant chemotherapy in the pancreatic tumour microbiome, Nalluri *et al*.^46^ (2021, USA)	1	1	2	1	5
Endoscopic ultrasound-guided fine-needle biopsy as a tool for studying the intra-tumoral microbiome in pancreatic ductal adenocarcinoma: a pilot study, Chu CS *et al*.^47^ (2022, China)	1	1	2	1	5
Analysis of the pancreatic cancer microbiome using endoscopic ultrasound–guided fine-needle aspiration–derived samples, Nakano S^48^ *et al*. (2022, Japan)	1	1	2	1	5
Impact of neoadjuvant therapy on gut microbiome in patients with resectable/borderline resectable pancreatic ductal adenocarcinoma, Takaori A *et al*.^49^ (2023, Japan)	1	1	2	1	5
Bacterial and fungal characterization of pancreatic adenocarcinoma from Endoscopic Ultrasound guided Biopsies, Wright *et al*.^50^ (2023, USA)	1	1	2	1	5
Gut Streptococcus is a microbial marker for the occurrence and liver metastasis of pancreatic cancer, Yang J *et al*.^51^ (2023, China)	1	1	2	1	5

^a^ Q1: Does the patient(s) represent(s) the whole experience of the investigator? Q2: Was the exposure adequately ascertained? Q3: Was the outcome adequately ascertained? Q4: Were other alternative causes that may explain the observation ruled out? Q7: Was follow-up long enough for outcomes to occur? Q8: Is the case(s) described with sufficient details to allow other investigators to replicate the research or to allow practitioners make inferences? (Questions 5 and 6 are relevant to cases of adverse drug events and were excluded)

### Characteristics of the studies

**Figure 2:**
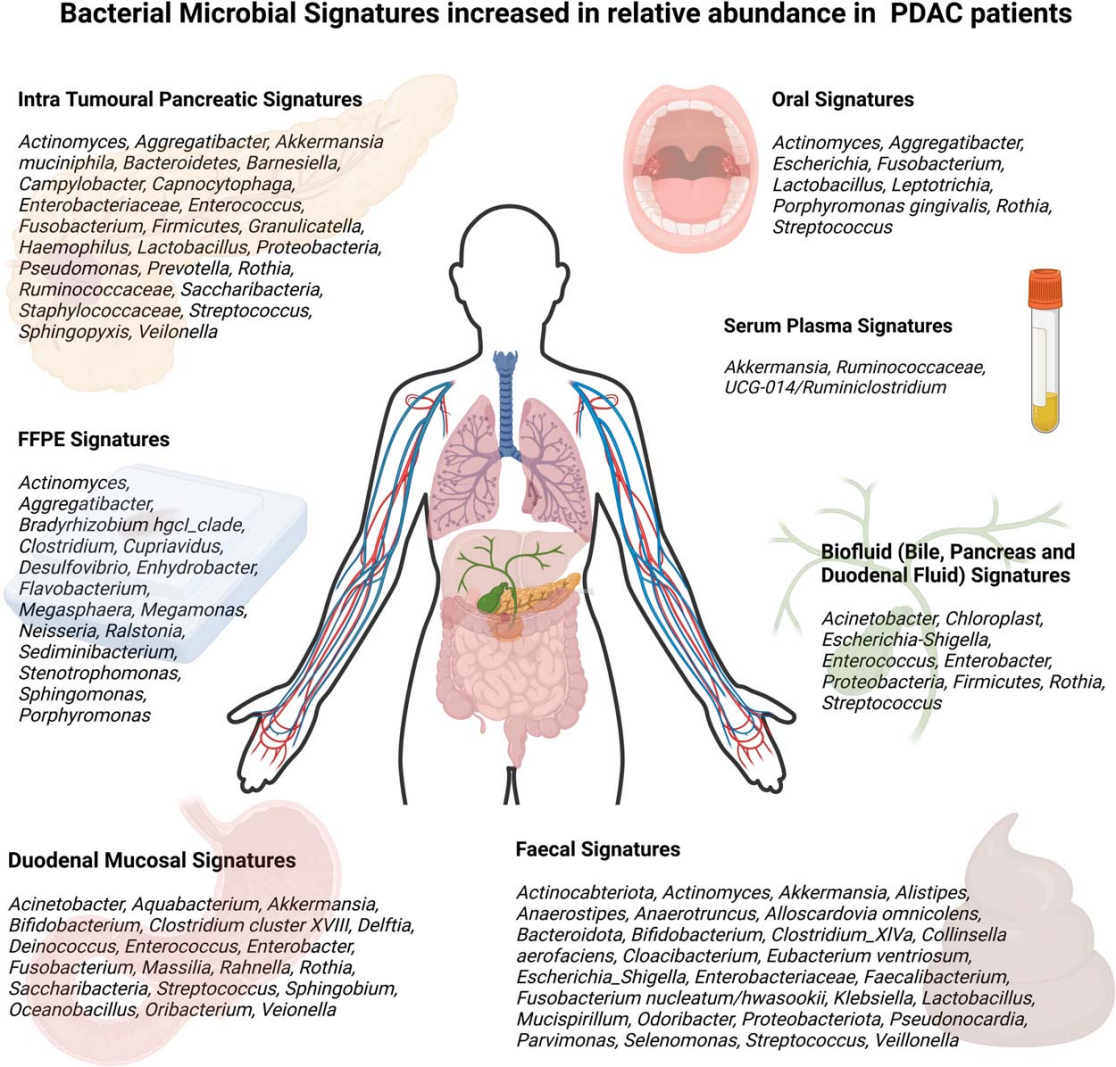
Summary of bacterial constituents in various compartments—oral, duodenum, faeces, bile, intra-tumoral, serum plasma, and FFPE tissue samples—with an elevated relative abundance compared to controls and other hepatopancreatobiliary disease states.

The full details of each selected study are separated into bodily compartments and bacteria found to be elevated in PDAC patients compared to healthy controls can be seen in Table 2. A summary of bacterial constituents of the microbiome that have been found to have increased relative abundance in PDAC is summarised in Figure 2.

Table 3: summary of included studies that used the next-generation technique, 16S rRNA gene amplicon sequencing to analyse compartment-specific microbiome changes in PDAC. Several studies have investigated more than one bodily compartment and are included more than once under the respective bodily compartment heading.

**Table 3:** Summary of included studies.

Article	Methodology	Sequencing and annotation	PDAC Signatures	Healthy and positive control signatures	Conclusions	Available Bio-data
Serum Plasma						
Microbiome Markers of Pancreatic Cancer Based on Bacteria-Derived Extracellular Vesicles Acquired from Blood Samples: A Retrospective Propensity Score Matching Analysis. Kim JR et al.^33^ 2021. South Korea	PDAC (*n*=38) Healthy controls (*n*=52) Microbial EVs via blood plasma.	16S rRNA gene analysis performed. V3-V4 hypervariable regions of the 16SrRNA gene. Taxonomic assignment was performed using UCLUST and QIIME against the GREENGENES reference database.	At the phylum level, most abundant: *Verrucomicrobia* *Deferribacteres* *Bacteroidetes* At the genus level, most abundant: *Akkermansia* *Ruminococcaceae* *UCG-014/Ruminiclostridium*	Most abundant in the control group Sphingomonas Propionibacterium Corynebacterium	These microbiome markers, which altered microbial compositions, are therefore candidate biomarkers for early diagnosis of PDAC.	Not available
Oral saliva						
Metagenomic identification of microbial signatures predicting pancreatic cancer from a multinational study. Nagata N *et al*.^42^ 2022. Japan	Japan cohort PDAC (*n*=47) Controls (*n*=235) Spanish cohort: PDAC (*n*=43) Controls (*n*=45) Saliva samples collected from patients with treatment-naïve PDAC and non-PDAC controls in Japan and Spain	16S rRNA gene analysis performed V3-V4 hypervariable regions of the 16SrRNA gene. The reads in the generated clusters were sorted by redundancy, and clustered with 97% identity using UCLUST. The 16S database was reconstructed from three publicly available databases: Ribosomal Database Project v.10.27 and a reference genome sequence database obtained from the NCBI FTP site.	Significantly enriched in the phylum of PDAC patients were: *Firmicutes (unknown Firmicutes, Dialister, and Solobacterium spp.)* *Prevotella spp. (P. pallens and P. sp. C561)*	Moreover, no oral species or genes were significantly different between patients with PDAC and controls. However, the following bacteria were depleted in PDAC cases: *Streptococcus. salivarius* *S. thermophilus* *S. australis*	The significant depletion of S. salivarius, which has been reported to have anti-inflammatory effects, was the most prominent signature in the PDAC oral microbiome.	Yes, available. https://www.sciencedirect.com/science/article/pii/S0016508522003547
A faecal microbiota signature with high specificity for pancreatic cancer. Kartal E *et al*.^39^ 2022 Germany, Spain	Spanish case–control PDAC (*n*=59) Controls (*n*=55) Oral Saliva, tissue and faecal samples were collected To account for potential bacterial contamination of extraction, negative controls (extraction blanks) were included.	16S rRNA gene analysis performed V3-V4 hypervariable regions of the 16SrRNA gene. Raw reads were quality trimmed and filtered against chimeric PCR artefacts using DADA2.	Data showed overall 27 enhanced levels of oral-intestinal transmission. *Veillonella spp*, were highly prevalent in both salivary (100% of subjects) and faecal (87.5%) samples across the entire study population.	Not mentioned	Faecal metagenomic classifiers performed much better than saliva-based classifiers and identified patients with PDAC based on a set of 27 microbial species. Significantly increased levels of oral-intestinal strain transmission in patients with PDAC.	Yes, available. PRJEB38625. PRJEB42013.
Comparisons of oral, intestinal, and pancreatic bacterial microbiomes in patients with pancreatic cancer and other gastrointestinal diseases. Chung, M *et al*.^45^ 2021. USA	PDAC (*n*=24) Ampullary adenocarcinoma (*n*=8) Cholangiocarcinoma (*n*=4) Benign controls (*n*=16) 316 oral samples (52 tongue swab, 46 buccal swab, 35 supragingival swab, 48 saliva samples) 6 normal pancreatic tissues 33 pancreatic tumour samples 22 Duodenum tissue 34 jejunum swab, 19 bile duct swab samples, 21 pancreatic ducts,	16S rRNA gene analysis performed V3-V4 hypervariable regions of the 16SrRNA gene. Sequence quality checking and denoising were performed using the DADA2 Illumina sequence denoising process. Taxonomic classification, alignment, and phylogenetic tree building were completed using QIIME2.	Bacterial communities from tongue and saliva samples clustered together, while those from buccal and supragingival samples formed another cluster. The saliva bacteria samples were: *Capnocytophaga gingivali* *Fusobacterium nucleatum* *Streptococcus ASVs.* Matched bacteria present between saliva and any pancreatic tissue were: *Fusobacterium* *Rothia* *Saccharibacteria* *Oribacterium* *Streptococcus*	Not mentioned	Findings indicate that oral, intestinal, and pancreatic bacterial microbiomes overlap but exhibit distinct co-abundance patterns in patients with pancreatic cancer and other gastrointestinal diseases.	Yes, available. PRJNA558364.
Dysbiotic gut microbiota in pancreatic cancer patients form correlation networks with the oral microbiota and prognostic factors. Matsukawa, H *et al*.^35^ 2021. Japan	PDAC (*n*=24) Healthy controls (*n*=18) 15 PDAC salivary samples were obtained.	16S rRNA gene analysis performed V3-V4 hypervariable regions of the 16SrRNA gene. Downstream sequences were processed using MacQIIME v1.9.1. Representative sequence taxonomies were assigned using the Greengenes reference database.	Multiple salivary microbes were present in the co-occurrence network. *Microbacterium* *Stenotrophomonas* These bacteria formed a network with faecal microbes in PDAC tissues.	Not mentioned	The dysbiotic gut microbiota in the pancreatic cancer patients forms a complex network with the oral and cancerous microbiota, and gut microbes abundant in these patients are related to poor overall survival.	Yes, available. PRJNA665854. PRJNA665618.
Characterization of Oral Microbiome and Exploration of Potential Biomarkers in Patients with Pancreatic Cancer. Sun, HY *et al*.^29^ 2020. China	PDAC (*n*=10) Benign pancreatic disease (BPD) (*n*=17) Healthy controls (HC) (*n*=10) 37 saliva samples	16S rRNA gene analysis performed V3-V4 hypervariable regions of the 16SrRNA gene. Paired-end reads into a single sequence by means of using FLASH software v.1.2.10. Then, 16S rRNA operational taxonomic units (OTUs) were selected from the combined reads via QIIME toolkit v.1.9.1.	The dominant bacteria in the PDAC group were: *Fusobacterium* *Megasphaera* *Prevotella* *Spirochaeta* *Treponema*	The dominant bacteria in the HC group (45.60%) and the BPD group (29.40%) were *Proteobacteria.* The dominant bacteria in the HC group were *Neisseriaceae*.	High concentrations of *Fusobacterium periodonticum* and low concentrations of *Neisseria* mucosa as specific risk factors for PDAC.	Yes, available. SRP237984.
Oral microbiome and pancreatic cancer. Wei, AL *et al*.^31^2020. China	PDAC (*n*=41) Healthy controls (*n*=69) Prior to surgery saliva samples were collected.	16S rRNA gene analysis performed V3-V4 hypervariable regions of the 16SrRNA gene. Raw sequences were denoised via FLASH. Quality filtering was performed on raw sequences using QIIME (v1.9.1) then high-quality clean tags were obtained. Tags were compared with gold database and chimeras were removed with the UCHIME algorithm (v11.0).	Compared with the healthy control group, carriage of *Streptococcus* and *Leptotrichina* was associated with a higher risk of PDAC. Among the patients with PDAC, patients reporting bloating have a higher abundance of: *Porphyromonas* *Fusobacterium* *Alloprevotella* While patients reporting jaundice had a higher amount of *Prevotella*.	*Veillonella* and *Neisseria* were considered a protective microbe that decreased the risk of PDAC and abundant in the healthy control group	Saliva microbiome was able to distinguish patients with PDAC and healthy individuals. Higher *Streptococcus* and *Leptotrichia* abundances were associated with increased risk of PDAC.	Yes, available. PMC7789059.
Oral microbial community composition is associated with pancreatic cancer: A case-control study in Iran. Vogtmann, E *et al*.^30^ 2020. USA, Iran	PDAC (*n*=273) Controls (*n*=285)	16S rRNA gene analysis performed V4 region of the 16SrRNA gene Sequence data processing was performed with QIIME 2 2017.2. Sequences quality control was performed with DADA2. Taxonomy was assigned to the Human Oral Microbiome Database version 14.51.	Increased risk of PDAC were associated with: *Enterobacteriaceae* *Lachnospiraceae G7* *Bacteroidaceae* *Staphylococcaceae* *Aggregatibacter actinomycetemcomitans*	An increased relative abundance of *Haemophilus* and *Proteobacteria*, were associated with decreased odds of PDAC.	The overall microbial community appeared to differ between pancreatic cancer cases and controls.	Yes, available. PRJNA549488.
Human oral microbiome and prospective risk for pancreatic cancer: a population-based nested case-control study. Fan, XZ *et al*. 2016. USA	PDAC (*n*=361) Controls (*n*=371) Population-based nested case–control study	16S rRNA gene analysis performed V3-V4 hypervariable regions of the 16SrRNA gene. Multiplexed and barcoded sequences were deconvoluted using the default parameters of the QIIME script split_libraries.py. Taxonomy was assigned to the Human Oral Microbiome Database version 14.51.	Oral pathogens associated with increased risk of PDAC: *Porphyromonas gingivalis* *Aggregatibacter actinomycetemcomitans* *Alloprevotella*	*Fusobacteria* and its genus *Leptotrichia* were associated with decreased risk of PDAC and common in the controls.	This study provides supportive evidence that oral microbiota may play a role in the aetiology of pancreatic cancer.	Not available
Characterization of the salivary microbiome in patients with pancreatic cancer. Torres, PJ *et al*.^25^ 2015. USA	PDAC (*n*=8) Positive controls (*n*=78) Healthy controls (*n*=22) Identified as contaminants were removed from all subsequent analyses.	16S rRNA gene analysis performed V3-V4 hypervariable regions of the 16SrRNA gene. 16S rRNA sequences were de-multiplexed using QIIME (v.1.8.0) pipeline. Sequences were grouped into OTUs at 97% sequence similarity using the Greengenes reference database.	At the phylum level, PDAC patients had higher proportions of *Firmicutes* and lower proportions of *Proteobacteria* At finer taxonomic levels, there was a higher proportion of *Leptotrichia* in PDAC patients.	*Porphyromonas* and *Neisseria* were lower in PDAC patients compared to the controls.	Bacteria abundance profiles in saliva are useful biomarkers for pancreatic cancer though much larger patient studies are needed to verify their predictive utility.	Yes, available. DNA Deposition file
Biofluid (Bile, Pancreas, and Duodenal Fluid)						
Bile Microbiome Signatures Associated with Pancreatic Ductal Adenocarcinoma Compared to Benign Disease: A UK Pilot Study. Merali N *et al*.^15^ 2023. UK	PDAC (*n*=12) Benign control group (*n*=17) ERCP performed to obtain bile samples.	16S rRNA gene analysis performed V3-V4 hypervariable regions of the 16SrRNA gene. The reads were quality checked by DADA2 (v1.25.2) R package [27214047]. Next, by using the “assignTaxonomy” function of DADA2, the reads were mapped to the formatted GTDB database.	In the same samples, the genus *Streptococcus* (FDR = 0.033) had increased abundance in the PDAC group.	Authors found three genera to be of significantly lower abundance among PDAC samples compared to benign group: *Escherichia* *Proteobacteria* *Enterobacteriaceae*	This study has demonstrated that patients with obstructive jaundice caused by PDAC have an altered microbiome in the bile compared to those with benign disease	Yes, available. PRJNA1018343.
Microbiomic profiles of bile in patients with benign and malignant pancreaticobiliary disease. Shyam K et al. 2023. USA	PDAC (*n*=25) Cholangiocarcinoma (*n*=6) Gallbladder cancer (*n*=1) Benign control group (*n*=14) ERCP performed to obtain bile samples.	16S rRNA gene analysis performed V3-V4 hypervariable regions of the 16SrRNA gene. After the sequencing, the paired-end sequences were processed with QIIME2 package (version 2019.7). The DADA2 pipeline within QIIME2 was used to trim the sequences, dereplicate, filter chimeric sequences and finally merge the paired end reads.	PDAC patients exhibited a predominance of genus *Rothia*. At the genus level, most abundant in PDAC were: *Dickeya* *Eubacterium hallii group* *Bacteroides* *Faecalibacterium* *Escherichia-Shigella* *Ruminococcus*	Cholangiocarcinoma showed a predominance of genera of: *Akkermansia* *Achromobacter*	Microbiome analyses of bile may differentiate malignant from benign samples in pancreaticobiliary diseases.	Yes, available. https://github.com/poudelmd/BileMicrobiome.
Biliary tract microbiota similarities in pancreatic ductal adenocarcinoma. Arteta.A et al. 2022. Colombia	PDAC (*n*=11) Benign control (*n*=3) Bile collected from the gallbladder as well as brushings from the intrapancreatic bile duct in PDAC cases.	16S rRNA gene analysis performed V3-V4 hypervariable regions of the 16SrRNA gene. Fastq files were analysed using Qiime2-2019. The analysis pipeline includes Dada2 for sequence quality control and an in-house trained classifier based on the Greengenes database for taxonomic analysis using a Qiime2 feature-classifier.	In both PDAC groups (GB and PD samples) predominant phylum were: *Proteobacteria (64-76%)* *Firmicutes (14-25%)* *Bacteroidetes (5-6%)* At class taxonomic level, *Gammaproteobacteria* represents 73% in PDAC.	Not mentioned	Compares microbiota using 16S rRNA in two anatomic locations of the biliary tract in PDAC patients (bile and biliary tract brush over pancreatic tumour.	Not available
Gallbladder microbiota composition is associated with pancreaticobiliary and gallbladder cancer prognosis. Kirishima M *et al*.^40^ 2022. Japan	PDAC (*n*=77) Cholangiocarcinoma (*n*=99) Gallbladder cancer (*n*=12) Pancreatic cyst (*n*=27) Benign other (*n*=29) Microbiome-derived DNA from the bile juice in surgically resected gallbladders.	16S rRNA gene analysis performed V3-V4 hypervariable regions of the 16SrRNA gene. Raw reads obtained from the sequencer were filtered according to the barcode and primer sequences using the MiSeq system. Then, the reads were imported into QIIME2 v2019.4 in Linux. Quality assessment, filtering, and chimera detection were performed using the DADA2 pipeline. Taxonomic classification was assigned to amplicon sequence variants using 99% clustering in SILVA 132 database.	Following bacteria showed a significant difference between with and without lymph node metastasis in PDAC: *Enterobacter* *Hungatella* *Mycolicibacterium* *Phyllobacterium* *Sphingomonas* Good prognosis factors for PDAC were: *Enterococcus* *Staphylococcus* *Bacteroides*	In the bile duct lesions, high relative abundance and poor prognosis were: *Enterococcus*, *Corynebacterium* *Haemophilus* *Lawsonella* *Staphylococcus* The microbiota in the normal gallbladder consists of main phyla: *Proteobacteria* *Firmicutes* *Bacteroidetes*	This study shows a link between gallbladder microbiota and pancreaticobiliary cancer prognosis.	Not available
Characteristics of bile microbiota in cholelithiasis, perihilar cholangiocarcinoma, distal cholangiocarcinoma, and pancreatic cancer. Li Z *et al*.^41^ 2022. China	PDAC (*n*=8) Cholangiocarcinoma (CCA) (*n*=23) Benign cholelithiasis (*n*=22) ERCP performed to obtain bile samples.	16S rRNA gene analysis performed V4 region of the 16SrRNA gene Amplified and processed using the QIIME2 platform and silva138.1 database was used to annotate species.	The significant 10 microbial biomarkers results for the PDAC group were: *Pseudomonas* *Chloroplast* *Acinetobacter* *Allorhizobium* *Neorhizobium* *Pararhizobium* *Rhizobium* *Exiguobacterium* *Halomonas* *Staphylococcus*	At genus level the biomarkers for proximal CCA were: *Pseudomonas* *Sphingomonas* *Halomonas* *Acinetobacter* *Prevotella* For distal CCA they were: *Streptococcus* *Prevotella* *Halomonas* *Helicobacter* *Rikenellaceae*	We found an increase in α diversity of the dCCA and PDAC groups compared to the benign group. As this pilot study identified specific microbial bile markers.	Not available
Alterations in the Duodenal Fluid Microbiome of Patients with Pancreatic Cancer. Kohi S *et al*.^11^ 2022. USA	PDAC (*n*=74) Pancreatic Cysts (*n*=98) Healthy controls (*n*=134) All patients underwent duodenal endoscopy	16S rRNA gene analysis performed V3-V4 hypervariable regions of the 16SrRNA gene. Raw sequences were analysed with QIIME2 2019.1. Raw sequence data were demultiplexed and quality filtered using DADA2. Taxonomy was assigned to ASVs using the SILVA (v132) database.	Duodenal fluid samples from patients with PDAC has higher levels of: *Escherichia-Shigella* *Enterococcus* *Clostridium sensustricto 1* *Bifidobacterium* PDAC with short-term survival patients had enrichment of: *Fusobacteria* *Rothia*	Duodenal fluid microbiome profiles were not significantly different between control subjects.	Patients with PDAC have alterations in their duodenal fluid microbiome profiles compared with patients with pancreatic cysts.	Yes, available. Bacterial PCR amplification sequencing with the supplementary file
Enterococcus spp. have higher fitness for survival, in a pH-dependent manner, in pancreatic juice among duodenal bacterial flora. Itoyama, S *et al*. 2021. Japan	PDAC (*n*=34). Duodenal or Bile duct cancer (BDC) (*n*=28) Pancreatic juice was collected after pancreatectomy from the drainage tube Only clear colourless pancreatic juice was used.	16S rRNA gene analysis performed V1-V2 hypervariable regions of the 16SrRNA gene. The paired‐end sequences obtained were merged, filtered, and denoised using DADA2. The taxonomic assignment was performed using the QIIME2 feature‐classifier plugin with the Greengenes 13_8 database.	*Enterococcus spp*. have a higher potential to survive and colonize in pancreatic juice than other bacteria in PDAC cases. The pancreatic juice of patients with PDAC and BDC has a highly heterogeneous bacterial composition.	Not mentioned	Alkalinity is one of the important factors for the selective survival of *E. faecalis* among microbiota.	Not available
Microbiome Patterns in Matched Bile, Duodenal, Pancreatic Tumour Tissue, Drainage, and Stool Samples: Association with Preoperative Stenting and Postoperative Pancreatic Fistula Development. Langheinrich, M *et al*.^44^ 2020. Germany	PDAC (*N*=10) Bile collected intra-operatively Tissue collected intraoperatively Preoperative collected faecal samples	16S rRNA gene analysis performed V3-V4 hypervariable regions of the 16SrRNA gene. Reads were demultiplexed and trimmed using Cutadapt, 16S the Uparse, and Sintax algorithms within Usearch using the silva 16S rRNA database (v123).	At genus level the most dominant genera within the bile fluid were: *Enterococcus* *Streptococcus* *Escherichia Shigella* *Veilonella* *Enterobacter*	Not mentioned	The microbiome is altered in patients undergoing preoperative stent placement. This cohort of patients have relatively more *Enterococci* in their bile, tumours, and duodenum.	Not available
Enrichment of oral microbiota in early cystic precursors to invasive pancreatic cancer. Gaiser RA *et al*.^43^ 2019. Sweden	PDAC (*n*=14) IPMN low grade (*n*=14) IPMN high grade (*n*=8) Paired cyst and plasma patients Plasma samples excluded due to low quality results.	16S rRNA gene analysis performed V1-V8 region (1381 bp) of the 16SrRNA gene These libraries were used as input for PacBio Single Molecule, Real-Time (SMRT) amplicon sequencing. Chimera sequences were filtered with UCHIME using a full length, good quality, and non-chimeric 16S rRNA gene reference database. To increase the granularity of the taxonomic assignment, sequences were also mapped to the HOMD 15.1 database.	Highly enriched in the PDAC group: *Methylobacterium* *Sphingomonas*	Cyst fluid from IPMN with high-grade dysplasia: Granulicatella Serratia Fusobacterium Cyst fluid from IPMN with low-grade dysplasia: Propionibacterium	The authors identified a co-occurrence and enrichment of oral bacterial taxa within the pancreatic cyst fluid samples. These findings warrant further investigation into the role of oral bacteria in cystic precursors to PDAC.	Not available
Formalin-fixed, paraffin-embedded (FFPE) tissue						
Intratumor Microbiome Analysis Identifies Positive Association Between Megasphaera and Survival of Chinese Patients with Pancreatic Ductal Adenocarcinomas. Huang, Y *et al*.^38^ 2022. China	PDAC (*n*=30) 13 short term survivors (OS <300 days) 17 long term survivors (OS>600 days) 16S rRNA gene analysis performed To eliminate any potential effect of contamination, same extraction, and sequencing procedures on margins of the paraffin blocks.	16S rRNA gene analysis performed V4 hypervariable regions of the 16SrRNA gene Each unique ASV was assigned to a high-resolution taxonomy using the Ribosomal Database Project classifiers (implemented in DADA2 pipeline) and SILVA Database v132.	*Megasphaera* specifically enriched in the LTS samples had a better inhibitory effect on tumour growth. LTS samples exhibited higher abundances of: *Sphingomonas* *Megasphaera* *Bradyrhizobium hgcI_clade* *Desulfovibrio* *Flavobacterium* *Enhydrobacter* *Megamonas* STS samples exhibited higher abundances of: *Clostridium-sensu stricto 1* *Actinomyces* *Porphyromonas* *Aggregatibacter* *Neisseria*	n/a	Patients with high relative abundances of *Sphingomonas* and *Megasphaera* were associated with significantly prolonged overall survival. High abundance of *Clostridium* were associated with shortened survival time.	Yes. Available PRJNA764032.
Dysbiotic gut microbiota in pancreatic cancer patients form correlation networks with the oral microbiota and prognostic factors. Matsukawa, H *et al*.^35^ 2021. Japan	PDAC (*n*=24) Healthy controls (*n*=18) FFPE tissue for PDAC patients Faecal samples: PDAC (*n*=24) Healthy controls (*n*=18)	16S rRNA gene analysis performed V3-V4 hypervariable regions of the 16SrRNA gene. Downstream sequences were processed using MacQIIME v1.9.1. Representative sequence taxonomies were assigned using the Greengenes reference database.	Genera were significantly abundant in PDAC tissues: *Sediminibacterium* *Microbacterium* *Ralstonia* *Stenotrophomonas* *Cupriavidus* *Microbacterium* and *Stenotrophomonas* were detected in PDAC tissues.	Not mentioned	The dysbiotic gut microbiota in the PDAC patients forms a complex network with the oral and cancerous microbiota, and gut microbes abundant in these patients are related to poor overall survival	Yes. Available PRJNA665854. PRJNA665618.
Endoscopic ultrasound (EUS)-guided fine needle biopsy (FNB) formalin fixed paraffin-embedded (FFPE) pancreatic tissue samples are a potential resource for microbiota analysis. Masi AC *et al*.^34^ 2021. UK	PDAC (*n*=8) Healthy controls (*n*=8) FFPE EUS-FNB samples were performed.	16S rRNA gene analysis performed V4 hypervariable regions of the 16SrRNA gene. Sequencing annotations not described.	Increase relative abundance of bacteria within PDAC: *Cloacibacterium* *Pseudomonas* *Corynebacterium* *Bacteroides*	There was an increase of relative abundance of the following bacteria within the healthy controls: *Streptococcus* *Tepidimonas* *Haemophilus* *Rothia*	There is potential of EUS-FNB FFPE samples to study the pancreas microbiome.	Not available
Tumour Microbiome Diversity and Composition Influence Pancreatic Cancer Outcomes. Riquelme, EM *et al*.^20^ 2019. USA	LTS PDAC, median survival 10.14 years (*n*=22) STS PDAC, median survival 1.62 years (*n*=21) FFPE of PDAC tissue were aseptically collected and bacterial genomic DNA was extracted.	16S rRNA gene analysis performed V4 hypervariable regions of the 16SrRNA gene. Raw paired-end 16S rRNA reads (V4 region) were merged into consensus fragments by FLASH and subsequently filtered for quality using QIIME. High-quality passing 16S rRNA sequences were assigned to a high-resolution taxonomic lineage using Resphera Insight and SILVA Database v128.	A higher alpha-diversity in the tumour microbiome of LTS patients. Intra-tumoral microbiome signature (Pseudoxanthomonas/Streptomyces/Saccharopolyspora/Bacillus clausii) highly predictive of long-term survivorship	n/a	PDAC microbiome composition can cross-talk with the gut microbiome, influences the host immune response and natural history of the disease.	Yes. Available PRJNA542615.
Duodenal mucosa tissue						
Comparisons of oral, intestinal, and pancreatic bacterial microbiomes in patients with pancreatic cancer and other gastrointestinal diseases. Chung, M *et al*.^45^ 2021. USA	PDAC (*n*=24) Ampullary adenocarcinoma (*n*=8) Cholangiocarcinoma (*n*=4) Benign controls (*n*=16) 22 Duodenum tissue 316 oral samples 34 jejunum swabs, 19 bile duct swab samples, 21 pancreatic ducts, 6 normal pancreatic tissues 33 pancreatic tumour samples.	16S rRNA gene analysis performed V4 hypervariable regions of the 16SrRNA gene. Sequence quality checking performed using DADA2. Taxonomic classification, alignment, and phylogenetic tree building were completed using the QIIME2 database.	Following bacteria were shown to be present between saliva, pancreatic or intestinal tissues of PDAC were: *Fusobacterium* *Rothia* *Saccharibacteria* *Oribacterium* *Streptococcus*	Not mentioned	Oral, intestinal, and pancreatic bacterial microbiomes overlap but exhibit distinct co-abundance patterns in patients with pancreatic cancer and other gastrointestinal diseases.	Yes, available. PRJNA558364.
Dysbiosis of the duodenal microbiota as a diagnostic marker for pancreaticobiliary cancer. Sugimoto, M *et al*.^36^ 2021. Japan	PDAC (*n*=12) Benign group (*n*=22) Endoscopic ultrasound-guided fine needle aspiration.	16S rRNA gene analysis performed V3-V4 hypervariable regions of the 16SrRNA gene. Bacterial classification was performed according to the OTUs, which were identified by correspondence to a database of human intestinal flora.	Useful biomarkers in PDAC and significantly different from the benign group were: *Clostridium cluster XVIII* *Bifidobacterium* *Prevotella*	The duodenal microbiota is more relevant to the pancreas and bile duct than is the salivary microbiota.	It was possible to investigate the microbiota of duodenal juice. Duodenal microbiota evaluation may contribute to the diagnosis of PDAC.	Not available
Microbiome Patterns in Matched Bile, Duodenal, Pancreatic Tumour Tissue, Drainage, and Stool Samples: Association with Preoperative Stenting and Postoperative Pancreatic Fistula Development. Langheinrich, M *et al*.^44^ 2020. Germany	PDAC (*n*=10) Bile collected intra-operatively Tissue collected intra-operatively Pre-operative collected faecal samples	16S rRNA gene analysis performed V3-V4 hypervariable regions of the 16SrRNA gene. Reads were demultiplexed and trimmed using Cutadapt, 16S the Uparse, and Sintax algorithms within Usearch using the silva 16S rRNA database (v123).	At genus level the most dominant genera within PDAC duodenal tissue group were: *Enterococcus* *Enterobacter* *Fusobacterium* *Akkermansia* *Veilonella*	n/a	The microbiome is altered in patients undergoing preoperative stent placement. This cohort of patients have relatively more *Enterococciin* their bile, tumours, and duodenum.	Not available
The Microbiomes of Pancreatic and Duodenum Tissue Overlap and Are Highly Subject Specific but Differ between Pancreatic Cancer and Noncancer Subjects. Del Castillo *et al*.^10^ 2019.USA	PDAC (*n*=51) Benign (chronic pancreatitis. Cysts)*n*=18 NDRI Organ donation (*n*=34) 189 tissue samples (pancreatic duct, duodenum, pancreas) 57 swabs (bile duct, jejunum, stomach) 12 stool samples To remove additional contamination, we removed a thin tissue layer around each sample prior to extracting DNA.	16S rRNA gene analysis performed V3-V4 hypervariable regions of the 16SrRNA gene. Sequences were BLASTN-searched against a combined set of 16S rRNA reference sequences that consist of the HOMD (version 14.5), Greengenes Gold, and the NCBI 16S rRNA reference sequence set. All assigned reads were subject to several down-stream bioinformatics analyses, including alpha and beta diversity assessments, provided in the QIIME software package version 1.9.1.	Significantly increased abundance in PDAC patients: *Fusobacterium spp.* Within the duodenal tissue of PDAC patients, *Selenomonas* was also elevated.	*Lactobacillus spp.* was significantly reduced in PDAC cancers compared with non-cancer patients	Bacterial DNA profiles in the pancreas were like those in the duodenum tissue of the same subjects. Suggesting that bacteria may be migrating from the gut into the pancreas.	Yes, available. PRJNA421501.
Characterization of the duodenal bacterial microbiota in patients with pancreatic head cancer vs. healthy controls. Mei, QX *et al*.^27^ 2018. China	PDAC (*n*=14) Healthy controls (*n*=14) Endoscopic duodenal mucosal biopsies.	16S rRNA gene analysis performed V3-V4 hypervariable regions of the 16SrRNA gene. OTUs were clustered with a 97% similarity cutoff using UPARSE (version 7.1) and chimeric sequences were identified and removed using UCHIME. The taxonomy of each 16S rRNA gene sequence was analysed using the RDP Classifier against the SILVA 119 16S rRNA database.	The most abundant bacteria at genus level were: *Acinetobacter* *Aquabacterium* *Oceanobacillus* *Rahnella* *Massilia* *Delftia* *Deinococcus* *Sphingobium*	Duodenal microbiotas of healthy controls were enriched with: *Porphyromonas* *Paenibacillus* *Enhydrobacter* *Escherichia* *Shigella* *Pseudomonas*	These results reveal a picture of duodenal microbiota in PDAC patients	Yes, available. SRP097254.
Intratumoural pancreatic tissue						
Bacterial and fungal characterization of pancreatic adenocarcinoma from Endoscopic Ultrasound guided Biopsies. Wright *et al*.^50^ 2023. USA	PDAC (*n*=15) 5 EUS FNA biopsies 10 unmatched surgical specimens	16S rRNA gene analysis performed V4 hypervariable regions of the 16SrRNA gene. High-quality passing 16S rRNA sequences were assigned to a high-resolution taxonomic lineage using Resphera Insight. High-quality passing ITS sequences were clustered into OTUs by UCLUST (de novo mode) and assigned a taxonomic lineage using the RDP classifier with the UNITE database.	EUS FNA identified PDAC bacteria: *Actinomyces* *Campylobacter* *Fusobacterium* *Granulicatella* *Haemophilus* *Prevotella* *Veilonella* *Prevotella* was the most abundant.	n/a	The Venn diagram and bar plot of genera composition observed at least 35 genera in common with 54% similarity between these two sample types.	Yes. Available. PRJNA1008674.
Endoscopic ultrasound-guided fine-needle biopsy as a tool for studying the intra-tumoral microbiome in pancreatic ductal adenocarcinoma: a pilot study. Chu CS *et al*.^47^ 2022. China	PDAC (*n*=9) 6 patients had EUS-FNB biopsy 4 patients had intraoperative biopsy (NB: 1 patient has both EUS FNB and open biopsy)	16S rRNA gene analysis performed V3-V4 hypervariable regions of the 16SrRNA gene. Quality-filtered and non-chimeric reads were analysed (UPARSE pipeline) to generate OTUs per sample (at 97% identity level). The OTU representative sequences were searched against the Greengenes 13_5 database by using USEARCH global alignment to identify the corresponding taxonomy.	The following bacteria were found to play a role in the development of PDAC: *Porphyromonas* *Fusobacterium* *Aggregatibacter* *Prevotella* *Capnocytophaga*	n/a	The intra-tumoral microbiome profile in tissues obtained by EUS-FNB had similar alpha-diversity and taxonomic profiles with those obtained by surgical biopsy.	Not available
Composition, diversity, and potential utility of intervention-naïve pancreatic cancer intratumoural microbiome signature profiling via endoscopic ultrasound. Gleeson FC *et al*.^19^ 2022. USA	PDAC (*n*=18) PNET (*n*=2) Acinar neuroendocrine carcinoma (*n*=1) Tissues were obtained via EUS fine needle biopsy.	16S rRNA gene analysis performed The hypervariable regions of the 16SrRNA gene were not mentioned. Sequencing annotations not described.	The predominant phyla were: *Proteobacteria* *Bacteroidetes* *Firmicutes* *Actinobacteria* *Gammaproteobacteria* (91%) and *Fusobacteriota* (38%) were the predominant genera	Three positive control tumours had relatively high *Helicobac*ter content, specifically *H. pylori*.	*Proteobacteria* dominated the microbiome in PDAC. No difference in either α-diversity or β-diversity metrics between anatomical locations or between the predominant phyla or genera were noted.	Not available
Analysis of the Pancreatic Cancer Microbiome Using Endoscopic Ultrasound–Guided Fine-Needle Aspiration–Derived Samples. Nakano S *et al*.^48^ 2022. Japan.	PDAC (*n*=30) Tissues were collected from patients who undergo EUS-FNA. 30 PDAC tissues matched with 30 duodenal and 30 stomach tissues (*n*=90)	16S rRNA gene analysis performed V3-V4 hypervariable regions of the 16SrRNA gene. Amplicon sequence analysis was performed using QIIME2 v.2020.8. Paired-end sequences were imported into QIIME2 and denoised using the DADA2 pipeline. Amplicon sequence variants were selected by aligning the sequences with those in the latest Silva 16S database (v.138).	There was a predominance of *Acinetobacter* and *Pseudomonas* in PDAC tissue. *Proteobacteria* were significantly more abundant in PDAC samples. *Delftia* was more abundant in resectable PDAC.	*Firmicutes, Bacteroidetes*, and *Fusobacteria* were significantly less abundant in PDAC tissues than in GI tissues.	PDAC tissues obtained by EUS-FNA were useful for analysing intratumor microbiome.	Not available
A faecal microbiota signature with high specificity for pancreatic cancer. Kartal E *et al*.^39^ 2022 Germany, Spain	Spanish case–control PDAC (*n*=23) Adjacent healthy tissue (*n*=20) Intraoperative tumour biopsies were taken To account for potential bacterial contamination of extraction, negative controls (extraction blanks) were included.	16S rRNA gene analysis performed V3-V4 hypervariable regions of the 16SrRNA gene. Raw reads were quality trimmed and filtered against chimeric PCR artefacts using DADA2.	Enriched in PDAC tissue were: *Lactobacillus spp* *Akkermansia muciniphila* *Bacteroides spp* FISH assays verified the prevalence of genus-specific primers in PDAC tissues: *Akkermansia spp* *Lactobacillus spp* *Bifidobacterium spp* *Veillonella spp* *Bacteroides spp*	Not mentioned	Several taxa could be traced between the gut and pancreas, with univariate enrichment in tumours relative to adjacent healthy tissue. Indicating direct associations of PDAC with the gut microbiome.	Yes, available. PRJEB38625. PRJEB42013.
Tumour microbiome contributes to an aggressive phenotype in the basal-like subtype of pancreatic cancer. Guo W *et al*.^12^ 2021. China	PDAC (*n*=62) PDAC tumour and adjacent tissues were collected intraoperatively	16S rRNA gene analysis performed V3-V4 hypervariable regions of the 16SrRNA gene. After sequence trimming and duplicate filtering, the passing reads were aligned to the human reference (GRCh38) using Bowtie2 to preliminarily remove the host DNA sequences. A customized database, which consists of reference libraries of bacteria, viruses, fungi, archaea, plasmids, UniVec and human from the NCBI database, was constructed for taxonomic classification in Kraken2.	Basal-like tumours had a distinct microbial community. Increasing abundance of: *Acinetobacter* *Pseudomonas* *Sphingopyxis*	n/a	These findings indicated that the tumour microbiome is closely related to PDAC oncogenesis and the induction of inflammation.	Yes, available. PRJNA719915
Role of biliary stent and neoadjuvant chemotherapy in the pancreatic tumour microbiome. Nalluri *et al*.^46^ 2021. USA	PDAC (*n*=27) PDAC tumour and adjacent tissues were collected intra-operatively	16S rRNA gene analysis performed V3-V4 hypervariable regions of the 16SrRNA gene. Amplicon sequence data were processed and analysed using Mothur software version 1.41.1. Reads were paired end joined, quality trimmed, and aligned against the SILVA database version.	Among both malignant and normal adjacent tissue samples, the most abundant families of bacteria include: *Ruminococcaceae* *Staphylococcaceae* *Bacillaceae* *Enterobacteriaceae* *Pseudomonadaceae.*	n/a	Preoperative biliary stent placement and neoadjuvant chemotherapy can encourage bacterial colonization of PDAC tissue.	Yes, available. SRP197553.
Comparisons of oral, intestinal, and pancreatic bacterial microbiomes in patients with pancreatic cancer and other gastrointestinal diseases. Chung M *et al*.^45^ 2021. USA	PDAC (*n*=24) Ampullary adenocarcinoma (*n*=8) Cholangiocarcinoma (*n*=4) Benign controls (*n*=16) 6 normal pancreatic tissues 33 pancreatic tumour samples 22 Duodenum tissue 316 oral samples (52 tongue swab, 46 buccal swab, 35 supragingival swab, 48 saliva samples) 34 jejunum swab, 19 bile duct swab samples, 21 pancreatic ducts,	16S rRNA gene analysis performed V3-V4 hypervariable regions of the 16SrRNA gene. Sequence quality checking and denoising were performed using the DADA2 Illumina sequence denoising process. Taxonomic classification, alignment, and phylogenetic tree building were completed using QIIME2.	*Fusobacterium nucleatum* was among the top shared species based on the taxonomic annotations between oral and intestinal or pancreatic samples. ASV (Amplicon Sequence Variants) that were present in PDAC were: *Fusobacterium* *Rothia* *Saccharibacteria* *Oribacterium* *Streptococcus*	Not mentioned	Oral, intestinal, and pancreatic bacterial microbiomes overlap but exhibit distinct co-abundance patterns in patients with PDAC and other gastrointestinal diseases.	Yes, available. PRJNA558364.
Microbiome Patterns in Matched Bile, Duodenal, Pancreatic Tumour Tissue, Drainage, and Stool Samples: Association with Preoperative Stenting and Postoperative Pancreatic Fistula Development. Langheinrich, M *et al*.^44^ 2020. Germany	PDAC (*N*=10) Bile collected intra-operatively Tissue collected intra-operatively Pre-operative collected faecal samples	16S rRNA gene analysis performed V3-V4 hypervariable regions of the 16SrRNA gene. Reads were demultiplexed and trimmed using Cutadapt, 16S the Uparse, and Sintax algorithms within Usearch using the silva 16S rRNA database (v123).	At genus level the most dominant genera within PDAC tissue group were: *Enterococcus* *Enterobacter* *Fusobacterium* *Barnesiella* *Akkermansia*	n/a	This study demonstrates that there is a distinct microbiome in the different compartments adjacent to the pancreas.	Not available
Faecal						
Gut Streptococcus is a microbial marker for the occurrence and liver metastasis of pancreatic cancer. Yang J *et al*.^51^ 2023. China	PDAC (*n*=44) Liver metastasis (LM*n*=27) Nonliver metastasis (non-LM,*n*=17) Healthy patients (*n*=50)	16S rRNA gene analysis performed V3-V4 hypervariable regions of the 16SrRNA gene. The data reads were filtered by the DADA2 method of QIIME2 software (v2019.4) and used to cross compare with the Greengenes database (release 13.8) for species annotation.	Significantly increased bacteria in PDAC were: *Streptococcus* *Lactobacillus* *Bifidobacterium* Linear discriminant analysis (LDA) was conducted to estimate the effect size (LEfSe) of each differential flora and found 16 significantly different microorganisms in the PDAC group.	11 significantly microbes in healthy group: *p_Bacteroidetes*, *c_Bacteroidia*, *o_Bacteroidales*, *f_Bacteroidaceae*, *g_Bacteroides*, *c_Clostridia*, *o_Clostridiales*, *f_Lachnospiraceae*, *g_Roseburia*, *g_Faecalibacterium*, *f_Veillonellaceae*	The study found that the intestinal microbial richness of PDAC patients was higher. The *Streptococcus* content was a predictive microbiota marker of PDAC (AUC of 0.927 (p < 0.001). As well as playing a key role in identifying liver metastases (AUC = 0.796, p < 0.001).	Yes. Available. PRJNA977486.
Impact of neoadjuvant therapy on gut microbiome in patients with resectable/borderline resectable pancreatic ductal adenocarcinoma. Takaori A *et al*.^49^ 2023. Japan	PDAC (*n*=20) Stool samples were collected from patients before and after neoadjuvant chemotherapy treatment (NAC). Faecal microbiota profiles before and after NAC were analysed using bacterial 16S rRNA gene sequences in patients with R/BR-PDAC.	16S rRNA gene analysis performed The hypervariable regions of the 16SrRNA gene were not mentioned. The sequence reads were imported into QIIME2 software and analysed for bacterial identification and diversity.	At the phylum level, *Firmicutes* was the most abundant bacteria before and after NAC. The next most common bacteria were: *Bacteroidota* *Actinocabteriota* *Proteobacteriota*	n/a	This study is the first to compare the gut microbiota before and after NAC for PDAC. Lower incidence of *Bifidobacterium* genus before NAC associated with a lower pathological response to NAC.	Not available
Changes in intestinal bacteria and imbalances of metabolites induced in the intestines of pancreatic ductal adenocarcinoma patients in a Japanese population: a preliminary result. Hashimoto S *et al*. 2022.Japan	Unresectable PDAC (*n*=5) Healthy controls (*n*=68)	16S rRNA gene analysis performed The hypervariable regions of the 16SrRNA gene were not mentioned. Sequencing annotations not described.	A significant increase in oral-associated bacteria were noted in PDAC cases: *Actinomyces* *Streptococcus* *Veillonella* *Lactobacillus*	A significant decrease of *Anaerostipes* was demonstrated in the faeces of PDAC patients compared with the control.	Showing the intestinal environment of PDAC patients is characterized by an increase in oral-associated bacteria.	Not available
Integrative analysis of metabolome and gut microbiota in patients with pancreatic ductal adenocarcinoma. Guo X *et al*. 2022^37^. China	Resectable PDAC (*n*=36) Unresectable PDAC (*n*=36)	16S rRNA gene analysis performed V3-V4 hypervariable regions of the 16SrRNA gene. The data was analysed with QIIME (version 1.9.1).	Resectable PDAC patients were: *Alistipes* *Anaerostipes* *Faecalibacterium* *Parvimonas* Unresectable PDAC patients were: *Pseudonocardia* *Cloacibacterium* *Mucispirillum* *Anaerotruncus*	n/a	There are metabolic and microbiome differences between resectable and unresectable PDAC patients.	Not available
A faecal microbiota signature with high specificity for pancreatic cancer. Kartal E *et al*.^39^ 2022 Germany, Spain	Spanish case–control faecal samples PDAC (*n*=51) Controls (*n*=46) Chronic pancreatitis (*n*=23) German case-control faecal samples PDAC (*n*=44) Controls (*n*=32)	16S rRNA gene analysis performed V3-V4 hypervariable regions of the 16SrRNA gene. Raw reads were quality trimmed and filtered against chimeric PCR artefacts using DADA2.	Enriched in faeces of patients with PDAC were: *Veillonella atypica*, *Fusobacterium nucleatum/hwasookii* *Alloscardovia omnicolens*	Whereas following bacteria species were depleted in PDAC: *Romboutsia timonensis* *Faecalibacterium prausnitzii* *Bacteroides coprocola* *Bifidobacterium bifidum*	The presented PDAC-specific microbiome signatures, including links between microbial populations across tissues, provide novel microbiome-related hypotheses.	Yes, available. PRJEB38625. PRJEB42013.
Metagenomic identification of microbial signatures predicting pancreatic cancer from a multinational study. Nagata N *et al*.^42^ 2022. Japan	Japan cohort: PDAC (*n*=47), Controls (*n*=235) Spanish cohort: PDAC (*n*=57), Controls (n=50) German cohort: 44 PDAC (*n*=44), Controls (*n*=32) Multinational shotgun metagenomic analysis of faecal samples collected from patients with treatment-naïve PDAC and non-PDAC controls in Japan, Spain, and Germany.	16S rRNA gene analysis performed V3-V4 hypervariable regions of the 16SrRNA gene. The 16S database was reconstructed from three publicly available databases: Ribosomal Database Project v.10.27 and a reference genome sequence database obtained from the NCBI FTP site.	Significant enrichments of gut signatures for PDAC in all the 3 cohorts: *Streptococcus* *Veillonella spp*	*Faecalibacterium prausnitzii* was consistently decreased in the gut microbiome of patients with PDAC in all the 3 cohorts.	The identification of shared gut microbial signatures for PDAC in Asian and European cohorts indicates the presence of robust and global gut microbial biomarkers	Yes, available. https://www.sciencedirect.com/science/article/pii/S0016508522003547
Dysbiotic gut microbiota in pancreatic cancer patients form correlation networks with the oral microbiota and prognostic factors. Matsukawa, H *et al*.^35^ 2021. Japan	PDAC (*n*=24) Healthy controls (*n*=18) Faecal samples: PDAC (*n*=24) Healthy controls (*n*=18) 16S rRNA gene analysis performed	16S rRNA gene analysis performed V3-V4 hypervariable regions of the 16SrRNA gene. Downstream sequences were processed using MacQIIME v1.9.1. Representative sequence taxonomies were assigned using the Greengenes reference database.	Four faecal microbes associated with poor survival in PDAC: *S. thermophiles*, *Bifidobacterium animalis* *Eubacterium ventriosum* *Collinsella aerofaciens*	Not mentioned	The dysbiotic gut microbiota in the PDAC patients forms a complex network with the oral and cancerous microbiota. Gut microbes abundant in these patients are related to poor overall survival	Yes, available. PRJNA665854. PRJNA665618.
Metataxonomic and Metabolic Impact of Faecal Microbiota Transplantation from Patients with Pancreatic Cancer into Germ-Free Mice: A Pilot Study. Genton, L *et al*.^32^ 2021. Switzerland.	Faecal microbiome transplant treatment PDAC (*n*=5) Healthy controls (*n*=5) Faecal samples collected within 7 days of recruitment	16S rRNA gene analysis performed V3-V4 hypervariable regions of the 16SrRNA gene. For the sequence analysis, paired reads were quality filtered using PEAR v0.9.11. Merged sequence reads were clustered using UNOISE3 from the USEARCH v10.0.240 pipeline and OTUs were classified using EzBioCloud 16S database.	PDAC was associated with: *Escherichia coli* *Streptococcus salivarius* *Enterobacteriaceae* *Proteobacteria*	These species were lower in PDAC patients and in mice transplanted with the faeces from these patients: *Alistipes obesi* *Lachnospiraceae* *Coriobacteriaceae*	The strengths of this study are its translational and innovative design, using the faeces of PDAC patients naïve of oncologic treatments and healthy volunteers.	Yes, available. PRJEB43581.
Microbiome Patterns in Matched Bile, Duodenal, Pancreatic Tumour Tissue, Drainage, and Stool Samples: Association with Preoperative Stenting and Postoperative Pancreatic Fistula Development. Langheinrich, M *et al*.^44^ 2020. Germany	PDAC (*N*=10) preoperative faecal sample Bile collected intra-operatively Tissue collected intra-operatively 16S rRNA gene analysis performed	16S rRNA gene analysis performed V3-V4 hypervariable regions of the 16SrRNA gene. Reads were demultiplexed and trimmed using Cutadapt, 16S the Uparse, and Sintax algorithms within Usearch using the silva 16S rRNA database (v123).	The predominant genera in the gut were: *Bacteroides* *Escherichia_Shigella* *Clostridium_XlVa* *Faecalibacterium* *Enterobacter*	n/a	This study demonstrates that there is a distinct microbiome in the different compartments adjacent to the pancreas.	Not available
Faecal microbiome signatures of pancreatic cancer patients. Half, E *et al*.^28^ 2019. Israel	PDAC (*n*=30) Nonalcoholic fatty-liver disease (NAFLD) (*n*=16) Precancerous lesions (PCL) (*n*=6) Healthy cohort (*n*=13) faecal samples collected after diagnosis and before treatment	16S rRNA gene analysis performed V3-V4 hypervariable regions of the 16SrRNA gene. Demultiplexed raw sequences were quality and merged using PEAR. Data was then processed with the QIIME package and VSEARCH, and according to the strategy described in the UPARSE pipeline. Taxonomy assignment used the UCLUST algorithm against Silva v128 database.	In this study we find a distinct PDAC-associated gut microbiome signature in an Israeli cohort: *Veillonellaceae* *Akkermansia* *Odoribacter*	Prevalent in the healthy control: *Clostridiacea* *Lachnospiraceae* *Ruminococcaceae* *Megasphaera* and *Lachnospiraceae UCG_008*, both of which were overrepresented in NAFLD as well as in PDAC. The genus *Veillonella* was associated with biliary obstruction.	The low incidence of PDAC and the high variability in microbiome both within and between the cohorts, harnessing microbial patterns for diagnostic purposes may only be practical if combined with additional biomarkers.	Yes, available. PRJNA575620
Gut microbial profile analysis by MiSeq sequencing of pancreatic carcinoma patients in China. Ren, ZG *et al*.^26^ 2017. China	PDAC (*n*=85) Healthy controls (*n*=57)	16S rRNA gene analysis performed V3-V5 hypervariable regions of the 16SrRNA gene. The amplified reads were processed by FLASH version 1.2.10 and sequences were detected with UCHIME version 4.2.40 with 16S “golden standard” database. Annotation of taxonomy sequences were performed using RDP classifier version 2.6.	In PDAC pathogens included were: *Veillonella* *Klebsiella* *Selenomonas* LPS-producing bacteria were enriched including: *Prevotella* *Hallella* *Enterobacter*	Whereas in the healthy controls: *Coprococcu Clostridium IV* *Blautia* *Flavonifractor* *Anaerostipe*	The gut microbial profile was unique in PDAC, providing a microbial marker for non-invasive PDAC diagnosis.	Yes, available. PRJEB13286.

### Blood-derived microbial signatures

Bacterial extracellular vesicles (bEV) are nano-sized, lipid membrane-delimited particles filled with bacteria-derived components and molecules such as DNA and metabolites^52^. bEV are thought to have an important role in bacterial physiology and pathogenesis and bacteria-bacteria and bacteria-host interactions^52^. Kim JR *et al*.^33^ extracted bEVs from blood serum in 38 patients with PDAC and 52 healthy controls and performed 16S rRNA gene sequencing. This revealed a compositional difference in the microbiome between both groups and highlighted six species with greater abundance in PDAC patients (*Ruminococcaceae UCG-014, Lachnospiraceae NK4A136 group, Akkermansia, Turicibacter, Ruminiclostridium, and Lachnospiraceae UCG-001*)^33^. These findings are consistent with studies that analysed the faecal microbiome in PDAC and further highlight the potential of blood serum as a cancer biomarker for PDAC^28^. The authors concluded that blood serum from patients with chronic infections such as the species identified in this PDAC cohort, contains elevated levels of antibodies to the bacteria or virus that may contribute to the disease process. However, the manipulation of these vesicles for therapeutic and diagnostic purposes is still in its infancy^53^.

### Oral microbial signatures

Localised aggressive periodontitis (LAP) disease is a chronic inflammatory condition and is an independent risk factor for PDAC^54^. The mechanism by which oral microbiota reach the pancreas occurs via translocation from biliary or pancreatic ducts, or the systemic circulation^55^. Nine studies reported results from 16S rRNA sequencing of oral saliva samples^25,29–31,35,39,42,45,56^. The most common study design was a comparison of the oral saliva microbiome between PDAC patients and controls^25,30,31,35,39,42,56,57^. Other control groups included patients with ampullary adenocarcinoma, cholangiocarcinoma (CCA), and intraductal papillary mucinous neoplasm (IPMN)^58^. The most commonly amplified region in the sequencing methods reported was the hypervariable V3-4 region.

Each study sought to identify bacteria associated with PDAC in the oral microbiome. A species identified by three studies to be associated with an increased risk of PDAC was *Aggregatibacter actinomycetemcomitans* ^30,43,56^. This is an anaerobic bacterium which has historically been thought to be associated with LAP. Similarly, elevated levels of *Porphyromonas gingivalis*, another bacterium implicated in periodontitis, were associated with PDAC^56^. Furthermore, two studies identified that a higher ratio of *Leptotrichia*, a constituent of normal oral flora, was associated with PDAC^56,57^. Conversely, bacteria of the genera *Neisseria* were found to be in greater numbers in oral saliva samples from the healthy control group, so it was inferred that these bacteria were protective^25,29,31,58,59^. Some specific species that were identified were *Neisseria elongata* ^59^ and *Neisseria flaviscens* ^58^. In a study by Farrell *et al*.^59^, the authors identified two bacteria, *Neisseria elongate* and *Streptococcus mitis*, and the combination of the two bacterial biomarkers could distinguish cases with PDAC from controls with 96.4% sensitivity and 82.1% specificity. One study investigated the relationship between oral, intestinal, and pancreatic microbiota by collecting samples from each of these areas from every patient^45^. The findings indicate that the microbiota from these areas overlap but exhibit distinct co-abundance patterns in patients with PDAC and other GI diseases^45^. Two studies sought to identify whether the oral microbiome may be able to assist in the diagnostic process for IPMNs^43,58^. Results were conflicting, with one study identifying some differences in the oral microbiome which the authors felt may be useful for future diagnostic use^43^. However, in a similar study, no major differences were identified between PDAC, IPMN, and healthy controls^58^.

### Biofluid signatures

Eight papers analysed microbiota in biofluid (bile, pancreatic, and duodenal fluid) using 16S rRNA gene sequencing in various benign and malignant pancreaticobiliary diseases^11,15,40,41,44,60–62^.

Merali *et al*. investigated ERCP bile samples from 12 treatment naïve PDAC, 10 choledocholithiasis, 7 gallstone pancreatitis, and 2 primary sclerosing cholangitis patients. Variable regions (V3–V4) of the 16S rRNA genes of microorganisms present in the samples were amplified by PCR and sequenced^15^. The bile microbial beta diversity significantly differed between patients with PDAC vs. benign disease (Permanova p=0.0173) and the separation of PDAC from benign samples was seen through unsupervised clustering of Aitchison distance. The authors found three genera to be of significantly lower abundance among PDAC samples vs. benign, adjusting for false discovery rate (FDR). These were *Escherichia* (FDR=0.002) and two unclassified genera, one from *Proteobacteria* (FDR=0.002) and one from *Enterobacteriaceae* (FDR=0.011).^15^ In the same samples, the genus *Streptococcus* (FDR=0.033) was found to be of increased abundance in the PDAC group. These findings highlight that the identification of specific bacteria in the bile may potentially enable the detection and stratification of PDAC. Poudel *et al*.^63^ investigated the bile microbial signatures using bile obtained from ERCP in 46 patients with either PDAC, CCA, gallbladder cancer, or benign biliary tract pathology. A multivariate approach was taken to classify Operational Taxonomic Units (OTU), which indicated that malignant pancreaticobiliary disease can be differentiated from benign, by distinct microbial signatures in bile. Specifically, a predominance of the genera *Dickeya, Eubacterium hallii group, Bacteroides, Faecalibacterium, Escherichia-Shigella*, and *Ruminococcus-1* in samples from pancreaticobiliary malignancies as compared to benign disease^63^. The differences between microbial signatures in PDAC versus CCA were also explored. Five dominant phyla (*Firmicutes, Bacteroidetes, Proteobacteria, Fusobacteria*, and *Actinobacteria*) were reported, with relatively similar abundance between the two groups^63^. These authors did not directly compare PDAC with benign disease, but at the genus level, patients with PDAC exhibited a predominance of the *Rothia* genus compared to those with CCA. Bile samples in CCA had a predominance of genera *Akkermansia* and *Achromobacter* compared to PDAC^63^. These findings highlight a distinct difference in dysbiosis between pancreaticobiliary cancers and benign disease, but also between various malignancies of the pancreaticobiliary system.

Li *et al*.^41^ investigated ERCP bile samples from 53 patients with different hepato-pancreato-biliary tract diseases including cholelithiasis, perihilar (pCCA), and distal cholangiocarcinoma (dCCA), and PDAC. 16S rRNA gene analysis and next-generation sequencing characterised specific microbial markers for each disease. Based on the results of linear discriminant analysis effect size (LEfSe), the three most significant biomarkers for pCCA at a genus level were *Pseudomonas, Sphingomonas*, and *Halomonas;* for dCCA, *Streptococcus, Prevotella*, and *Halomonas*; and PDAC, *Pseudomonas, Chloroplast*, and *Acinetobacter* ^41^. There was an increase in alpha diversity in the dCCA and PDAC groups compared to benign controls. It should be noted that the study stated exclusion criteria which included receiving various medications within 1 month of the sample (such as antibiotics, proton pump inhibitors, and prebiotics) and previous chronic diseases such as malignancy. Many of the included studies did not control for these factors, which are known to confound the GI microbiome^40,44,60^.

Kirishima *et al*.^40^ describe conflicting results to Li *et al*. In 244 patients with CCA, PDAC, gallbladder cancer, pancreatic cysts. and a variety of benign inflammatory lesions, there was little to no microbiome difference between the type of lesion and anatomical location^40^. However, the authors sampled bile from surgically extracted gallbladders. This method of bile extraction may preclude comparison with studies using bile extracted at ERCP. Some of this cohort had received chemotherapy, but the authors did not state if patients had received any antimicrobials, therefore the lack of difference between groups is unaccounted for. The authors did suggest the potential use of microbial biomarkers in predictive prognostic strategies^40^. Adjusting for clinicopathological variables such as age, sex, ASA score, and evidence of lymph node invasion and neoadjuvant chemotherapy (NAC) in PDAC or CCA, allowed for the correlation of relative abundance of certain microbiota with clinical outcome. For example, *Enterobacter, Hungatella, Mycolicibacterium, Phyllobacterium*, and *Sphingomonas* had significantly different relative abundance between PDAC patients with and without lymph node metastasis^40^. Positive prognostic factors for PDAC were found to be *Enterococcus, Staphylococcus*, and *Bacteroides* ^40^. There was no common microbe correlated with a poor prognosis between the types of lesions. However, in bile duct lesions only, poor prognosis was associated with a high relative abundance of *Enterococcus, Corynebacterium, Haemophilus, Lawsonella*, and *Staphylococcus.* In the normal gallbladder, they also reported the microbiota consists of four main phyla: *Proteobacteria, Firmicutes*, and *Bacteroidetes* ^40^.

Arteta *et al*.^62^ performed 16S rRNA gene analysis on 20 bile samples extracted directly from the gallbladder in 13 patients with PDAC, at the time of surgery. They also analysed intrapancreatic bile duct brushings from the PDAC cohort. In both these groups, the predominant phyla were Proteobacteria (64–76%), *Firmicutes* (14–25%), and *Bacteroidetes* (5–6%) and at the class taxonomic level, Gammaproteobacteria represented 73% of the overall microbiome in PDAC^62^. By performing liquid chromatography and mass spectrometry, human, and bacterial proteomic profiles were characterised, and three bacterial infection pathways were over-represented in the human PDAC^8^. Based on these findings, the authors were able to compare the microbiota in PDAC using 16S rRNA sequencing in two anatomical locations of the biliary tract, proposing a bacterial-induced carcinogenesis model for the biliary tract^62^. One case–control study explored bacterial and fungal (18SrRNA) sequencing of duodenal fluid in 74 PDAC cases compared to 98 benign controls with pancreatic cysts. All patients underwent duodenal endoscopy, and the risk of contamination was examined by 16S rRNA gene analysis of sterile water from the endoscopes. There was enrichment of *Escherichia-Shigella, Enterococcus, Clostridium sensustricto 1*, and *Bifidobacterium* in PDAC compared to benign controls^11^. Furthermore, it was noted that there was a significant increase in *Rothia* and *Fusobacteria* in the duodenal fluid of PDAC patients with better short-term survival^11^.

Gaiser *et al*.^43^ 2019 investigated the potential intracystic pancreatic microbiome in a pancreatic cystic neoplasm (PCN) surgery patient cohort. IPMN are most common type of PCN and can be an early detection marker for PDAC^64^. Matched pancreatic cystic fluid and plasma blood samples were obtained from 105 patients for microbiota analysis. Paired cyst fluid and plasma were collected at pancreatic surgery from patients with suspected PCN (*n*=105)^43^. Unfortunately, the quality of plasma 16S DNA results was very low and therefore excluded from the analysis. The authors mentioned that this appeared to be a local (inflammatory potential of the pancreatic microbiome) rather than a systemic effect, as IPMN disease does not communicate with the pancreatic duct system, which drains into the gut. After full-length 16S rRNA gene sequencing and quality thresholds, cyst fluid samples from 35 patients classified as IPMN low-grade disease (*n*=14) or IPMN high grade disease (*n*=8) and PDAC (*n*=14) were subjected to microbiome analysis. The authors identified a co-occurrence and enrichment of oral bacterial taxa within the pancreatic cyst fluid samples. At phylum level, IPMN low-grade disease was found to be rather homogeneously dominated by *Proteobacteria* ^43^. IPMN high-grade diseases were highly enriched with genera *Granulicatella*, *Serratia*, and *F usobacterium* ^43^. Whereas the PDAC group were highly enriched with genera *Methylobacterium and Sphingomonas* ^43^. These findings warrant further investigation into the role of oral bacteria in cystic precursors to PDAC. As well as intracystic pancreatic fluid samples may represent a potential therapeutic strategy for patients associated with high-grade IPMN and PDAC.

A major limitation of the studies is the current lack of understanding of the healthy bile microbiome. Sampling of bile in healthy patients poses an ethical challenge, due to the invasive nature of ERCP or surgery. Molinero *et al*. attempted to overcome this by evaluating bile from liver donors without a history of biliary or hepatic disorders. They found an increase in sequences from the *Propionibacteriaceae* family versus healthy controls^61^. The lack of information relating to environmental, host, and tumour factors provided by these studies limits comparison. To our knowledge, no studies have established whether the method of bile sampling has implications for subsequent microbiome analysis. However, contaminants from the oral cavity, upper GI tract, or liver and skin flora seem likely to confound results. For example, there may be intestinal milieu contamination during ERCP collection, and it is difficult to ensure that bacteria of duodenal origin are excluded from the bile. It is also likely that direct contamination of the endoscope leads to the introduction of bacteria from the oral and oesophagogastric cavities. No studies to date analysing bile microbiome in PDAC have compared bile samples with oral, stomach, or duodenal fluid samples^61^. However, multiple studies have examined the faecal microbiome in various cancers, but we hypothesised that as the pancreas is an upper GI organ that perhaps the bile or duodenal fluid would be more appropriate for looking at the pancreatic cancer microbiome.

### Formalin-fixed paraffin-embedded pancreatic tissue signatures

Four studies reported results from 16S rRNA gene sequencing of archival FFPE PDAC samples. In a Chinese cohort of 13 short-term survivors (STS) (OS<300 days) and 17 LTS (OS>600 days), the impact of the intratumoral microbiome characteristics was correlated with progression^38^. This study identified higher intratumor microbiome diversity in LTS compared with STS and observed a higher abundance of *Proteobacteria*, *Firmicutes*, and *Bacteroidetes* for all samples^38^. The authors found that *Sphingomonas* and *Megasphaera* were highly enriched in LTS tumour tissues^38^. Further work showed that *Megasphaera* sp.*XA511* promotes the secretion of proinflammatory cytokines *in vitro* and enhances the efficacy of PD-1 blockade in vivo. Riquelme *et al*. found different dominant genera in their US cohort, with higher alpha-diversity in the tumour microbiome of LTS patients and an intratumoral microbiome signature (*Pseudoxanthomonas/Streptomyces/Saccharopolyspora/Bacillus clausii*) highly predictive of long-term survivorship in both discovery and validation cohorts^20^. Faecal microbiota transplantation *ex-vivo* from short-term and long-term donors, showed that modulation of the tumour microbiome was associated with tumour growth and immune infiltration^20^. A Japanese study identified a significant presence of *Microbacterium* and *Stenotrophomonas* in seven PDAC patients that formed co-occurrence networks with faecal, salivary, and tumour microbes and were closely linked with poor prognostic factors in the network^35^. Masi *et al*. demonstrated the feasibility of using pancreatic FFPE samples from EUS fine needle biopsy to study the pancreatic microbiome. They found an increased abundance of *Cloacibacterium, Pseudomonas, Corynebacterium*, and *Bacteroides* in PDAC samples^34^. FFPE archival tissues may provide a large resource of potential microbial data from patients, but the use of these low-biomass specimens remains challenging^65^. FFPE tissue samples are primarily driven by human DNA rather than bacterial DNA presence^65^. Recent research indicates that the paraffin embedding process may impact the microbial profile, as FFPE tissue samples are predominantly characterized by human DNA rather than bacterial DNA^66^. Despite the complexities associated with conducting 16S rRNA amplicon sequencing on FFPE tissue specimens, ensuring the inclusion of negative and positive controls is crucial for maintaining quality control^65^.

### Duodenal mucosa tissue signatures

The gut microbiome is key to the development and modulation of the mucosal innate and adaptive immune system and is known to influence the outcome of patients with cancer^67^. Studies have also tentatively linked the duodenal and pancreatic tissue microbiota with an increased risk of PDAC^10,27,36,44^. Chung *et al*.^45^ examined oral cavity, intestinal, and pancreatic tissue samples from pancreatic cancer patients. The authors showed a clear separation between oral bacterial communities and bacterial communities from duodenal tissue, bile duct swab, pancreatic duct, and pancreatic tissue samples. This suggests that bacteria may migrate through the digestive system between several anatomically distinct sites^45^. Langheinrich *et al*. characterised and compared different compartments (bile duct, duodenal mucosa, pancreatic tumour site, postoperative drainage fluid, and stool samples) both preoperatively and postoperatively in 10 patients undergoing pancreatic surgery^44^. This showed a distinct profile in each compartment and also a trend toward a higher abundance of *Enterococcus* in patients with pancreatic stent versus no stent placement (not reaching statistical significance)^44^. This is possibly related to retrograde bacterial colonisation of the stent via the duodenal compartment^44^. Sugimoto *et al*. investigated the accuracy of duodenal microbiota for diagnosing PDAC. *Clostridium* cluster XVIII proved to be more valuable, for the diagnosis of PDAC compared to the blood marker Carbohydrate antigen 19 9 (CA 19 9) and other bacterial species^36^. Notably, in combination *Clostridium* cluster XVIII and CA19-9 showed a high sensitivity of 91.7% and specificity of 71.4%, suggesting that this combination has utility in PDAC screening^36^. Del Castillo *et al*.^10^ used pancreatic and duodenal tissue from patients with pancreatic cysts or PDAC and compared to deceased pancreatic tissue samples (obtained from donors who died of noncancer causes), sequencing of 189 tissue samples (pancreatic duct, duodenum, and pancreas), 57 swabs (bile duct, jejunum, and stomach) and 12 stool samples. *Lactobacillus* was significantly higher in noncancer controls compared to PDAC, and the relative abundance of *Fusobacterium* spp. was higher in cancer compared to noncancer subjects^10^. The authors also demonstrated that the microbiome within the pancreas was similar to duodenal tissue from the same patient, regardless of disease state. Mei *et al*.^27^ studied the duodenal mucosal microbiota in 14 patients with PDAC and 14 healthy. PDAC showed diffuse duodenal mucosa inflammatory cell infiltration in the lamina propria with a higher frequency of *H. pylori* infection. The sequencing analysis showed that species in both groups belong mainly to the phyla *Firmicutes* and *Proteobacteria* ^27^. Overall, this may suggest that the bacterial environment of the duodenum is intimately related to that of PDAC.

### Intratumoural pancreatic tissue signatures

Studies have consistently suggested that there is a specific tumoral microbiome in PDAC, which influences carcinogenesis, response to chemotherapy, and prognosis^68^. As previously discussed, colonisation of the pancreas is thought to be due to migration from oral, GI, and hepatobiliary cavities^5,10,11,69^. The route of colonisation is debated, but direct reflux of bacteria from the duodenum into the pancreatic duct, and translocation from the lower GI tract into the hepato-portal circulation or mesenteric lymph nodes are anatomical explanations^70^. Geller *et al*.^13^ demonstrated the presence of *Gammaproteobacteria* might be responsible for PDAC tumour resistance to gemcitabine.

Wright *et al*.^50^ recently published a pilot study on the feasibility of assessing bacterial and fungal populations in five patients with PDAC via endoscopic ultrasound-guided fine-needle biopsy (EUS-FNB). The 16S rDNA region V4 was amplified by PCR and *Actinomyces, Campylobacter, Fusobacterium, Granulicatella, Haemophilus, Prevotella*, and *Veilonella* were identified in all five samples. *Prevotella* was the most abundant^50^. Comparing these results with 10 unmatched individual PDAC patients that had samples obtained at surgery, the Venn diagram and bar plot of genera composition observed at least 35 genera in common with 54% similarity between these two sample types^50^. The researchers have also described a proof-of-concept method that can characterise and conserve bacterial signatures using EUS-FNA biopsy before resectional surgery. Further investigation is needed with greater sample sizes, using mucosal tissue for normalisation, and matched tissues for validation. Chu *et al*.^47^ studied nine patients diagnosed with PDAC and generated intratumoral microbiome profiling from EUS-FNB, as well as surgical biopsy. This revealed that EUS-FNB was a valid tool for studying the intratumoral microbiome in patients with both resectable and unresectable PDAC^47^.

Gleeson *et al*. performed a prospective study of EUS-acquired fresh pancreatic tumour tissue from 18 treatment-naive patients^19^. At the genus level, the PDAC group were composed of *Paracoccus, Brevundimonas, Prevotella, Cutibacterium*, *and Streptococcus genera* ^19^. The authors found no difference in either α-diversity or β-diversity of the microbiome compared to tumour location. Similarly, Nakano S *et al*.^48^ analysed matched pancreatic tissue with duodenal and stomach tissues in 30 patients with PDAC undergoing EUS- fine needle aspiration. The authors found that intratumoral pancreatic tissue had a lower microbial diversity than stomach and duodenum tissue, and fewer bacteria were detected in the head or tail of the pancreas^48^. *Proteobacteria* were more abundant, whereas *Firmicutes, Bacteroidetes, and Fusobacteria* were less abundant in PDAC tissues than in stomach and duodenal tissue^48^. They also found no significant difference in the tumour bacterial diversity and composition at the phylum level between patients with resectable and unresectable disease^48^.

Guo *et al*.^12,71^ conducted a study that associated microbial communities with either classical or basal-like transcriptomic tumour subtypes (the latter being associated with poorer clinical outcomes). PDAC tumour and adjacent tissue were collected intraoperatively from 62 patients and distinctive microbial communities were identified in basal-like tumours, with increased *Acinetobacter, Pseudomonas*, *and Sphingopyxis* observed^12^. This argues for the predictive value of the microbiome via the subtype-dependent microbiome compositions, but these transcriptome-based subtypes have not been implemented into clinical practice. It has been demonstrated that one in six patients with PDAC have tumours that fail to reliably fall into classical or basal-like PDAC subtype categories, and a subset of PDAC tumours harbour a mix of basal-like and classical cell populations^72^. Nalluri *et al*.^46^ analysed matched PDAC tumours and normal pancreatic tissue from 27 patients who underwent surgery. A significantly higher relative abundance of *Enterobacteriaceae* was observed in patients who had undergone biliary stent placement, or neoadjuvant chemotherapy^46^. The researchers then postulated that migration of microbes from the biliary tract during biliary stenting, adjacent to the pancreas, may influence the growth of intratumour microbiota. In addition, the immunosuppressive effect of chemotherapy may expose patients to an increased risk of stenting and antibiotic use for stent-related cholangitis^46^.

Chung *et al*. correlated the microbiome in the oral cavity with the microbiome in the pancreatic tissue of the same patients. PDAC patients had higher levels of *Firmicutes* and several related taxa (*Bacilli, Lactobacillales, Streptococcaceae, Streptococcus, Streptococcus thermophilus*), although, results remained significant at the phylum level only^45^. However, an Amplicon Sequence Variants (ASV) corresponding to the genus *Streptococcus* was the only ASV showing a high probability (56%) to be present in both saliva and pancreatic tissue samples^45^.

Langheinrich *et al*.^44^ characterised and compared the microbial communities and texture (soft versus hard) of the pancreatic tissue in patients undergoing surgery for PDAC. Overall, the soft tissue group harboured more *Firmicutes*, while *Proteobacteria* and *Verrucomicrobia* were more abundant in the hard tissue group^44^. Neither bacterium was found to have significance in the risk of development of a postoperative pancreatic fistula (POPF). Interestingly, *Fusobacteria* were enriched in malignant PDAC tissues^44^. The microbiome shows characteristic changes in neoplastic diseases; the transformed microbiome, a characteristic of neoplasia, is termed the oncobiome^73^.

### Faecal microbial signatures

Eleven studies showed that gut microbiome was different between healthy controls and PDAC subjects^26,28,32,35,37,39,42,44,49,51,74^. Yang *et al*. analysed the faecal samples of 44 treatment naïve PDAC patients and 50 healthy patients. The PDAC patients were sub-divided into the liver metastasis group (LM group, *n*=27) and the nonliver metastasis group (non-LM group, *n*=17)^51^. DNA was extracted and 16S rRNA gene sequencing was performed. The study found that the intestinal microbial richness of PDAC patients was higher^51^. The *Streptococcus* content was significantly increased and was a predictive microbiota marker of PDAC (AUC of 0.927 (*P*<0.001)^51^ and identifying liver metastases (AUC = 0.796, *P*<0.001)^51^.

Takaori *et al*.^49^ analysed stool samples before and after neoadjuvant chemotherapy in 20 patients with resectable or borderline PDAC. The authors suggest that a lower incidence of *Bifidobacterium* genus before NAC may be associated with a poorer response^49^. Kartal *et al*.^39^ explored faecal and salivary microbiota as potential biomarkers, from a Spanish and German case–control study. The faecal metagenomic classifiers performed better than saliva. The authors discovered 27 microbial species that could be employed to identify PDAC with high accuracy, particularly in combination with CA 19-9^39^. In a multinational study, Nagata *et al*. conducted a comprehensive analysis by performing shotgun metagenomic analysis on faecal and salivary samples obtained from a Japanese cohort. Additionally, they reanalysed data from Kartal *et al*. involving 47 Japanese patients with treatment-naive PDAC and 235 controls without PDAC, the study revealed the presence of 30 gut bacterial species, 18 oral bacterial species, and 58 bacteriophages associated with PDAC^42^. This study demonstrated reproducible gut microbial and functional signatures across three diverse cohorts in different geographical locations. The gut species significantly associated with PDAC were *Veillonella* spp. (*V parvula* and *V atypica*) and *Streptococcus* spp. (*S anginosus* and *S oralis*)^42^. Furthermore, they identified 58 new phages that could infect these 4 microbial species^42^. Phage treatment is a promising approach to modulating the microbiome by eliminating only certain species, unlike broad-spectrum antibiotic treatment, which disrupts the entire structure of the microbiome and can lead to resistance and side effects^42^.

Matsukawa *et al*. investigated 24 PDAC patients and 18 healthy controls. Results showed the gut faecal microbiota of patients with PDAC to be both taxonomically and functionally altered from that of healthy controls^35^. In total, 26 species significantly differed between the two cohorts. It was also found that 44 Kyoto Encyclopaedia of Genes and Genomes (KEGG) metabolic pathways differed significantly between healthy controls and PDAC patients^35^. Univariate analysis showed the relative abundance of certain faecal microbiota (*Bifidobacterium animalis, Collinsella aerofaciens, Eubacterium ventriosum, K. pneumoniae, Roseburia intestinalis*, *and S. thermophilus)* was associated with an increased mortality hazard ratio^35^. However, no viruses, microbial pathways or salivary microbes were significantly associated with this. This study used 16S rRNA sequencing to assess PDAC tissue from seven of 24 patients who had undergone surgery. This found both *Microbacterium* and *Stenotrophomonas* formed co-occurrence networks with salivary and faecal microbes^35^. Despite the study’s limited number of patients, it successfully showcased species-level dysbiosis in the gut microbiota of PDAC patients. This dysbiosis formed an intricate network with oral and cancer microbiota and was linked to prognostic factors.

Ren *et al*. studied 85 patients with PDAC and 57 matched healthy controls. The patients with pancreatic cancer were further subdivided into 54 with cancer of the pancreatic head, and 31 with cancer of the pancreatic body and tail. The 54 pancreatic head cancers were then divided into both obstructed and non-obstructed common bile ducts^26^. PDAC patients were shown to have significantly decreased microbial diversity. When comparing pancreatic head and pancreatic body or tail microbiota, and obstructed and nonobstructed common bile ducts, no differences were observed. Faecal microbial communities in PDAC were different to healthy controls. *Bacteroidetes*, *Firmicutes*, and *Proteobacteria* were the dominant bacterial phyla in both PDAC and healthy controls, but *Bacteroidetes* was significantly increased in PDAC (*P*<0.001)^26^. *Firmicutes* and *Proteobacteria* were significantly decreased in PDAC (*P*<0.05)^26^. Half *et al*.^28^ analysed 30 patients with PDAC, 6 patients with precancerous lesions, a healthy control group of 13 patients, and 16 with nonalcoholic fatty liver disease (NAFLD). The ratio of *Bacteroidetes* to *Firmicutes* was higher in PDAC patients, however, the abundance of bacteria was lower. This was similar to findings in the controls with NAFLD. The genera examined in NAFLD, and healthy control groups showed similar trends, however, *Megasphaera* and *Lachnospiraceae* were over-represented in NAFLD controls and PDAC^28^. When assessing at the phylum level, PDAC patients had a higher incidence of *Bacteroidetes* and a decrease in *Firmicutes*. The data from this study was combined with data from the study by Ren *et al*.^26^ to explore inter-cohort similarities. This identified under-abundance of several bacteria, *Clostridiacea*, *Lachnospiraceae*, and *Ruminococcaceae*; and an over-abundance of *Veillonellaceae*, *Akkermansia*, and *Odoribacter*. This was distinct from the microbiome profile in individuals with complications of PDAC, including bile duct obstruction and liver injury. This study was limited by the low number of patients enroled and vast differences in demographic factors and method of DNA extraction for 16S rRNA sequencing.

Genton *et al*.^32^ examined the faecal microbiome in PDAC when compared to healthy controls. The faeces were then transplanted into two mice per case, DNA was extracted from the stool of the mice and the V3–4 region of the bacterial 16S rRNA genes was amplified. Comparing the microbiota of the faeces of the PDAC cases and healthy controls showed a marked abundance of *Escherichia coli* and *Streptococcus salivarius* in PDAC (>147-fold and >31-fold, respectively)^32^. The results also showed that the visceral fat of mice who received stool from PDAC patients was lower than mice who had received stool from controls. The study also showed the microbiota of transplanted mice reflects the taxonomic composition of the faeces from the human donor. The researchers conclude that the link between visceral fat, chronic inflammation cytokine activity, and carcinogenesis warrants further study. While this study included patients naïve to oncologic treatments, it was also limited in the number of PDAC patients and the duration of time that was afforded to monitor the change in murine models.

## Discussion

The human microbiome is increasingly investigated in both basic and translational science. The potential for modulation could be a future target for this new hallmark in cancer. The microbiota has been shown to regulate metabolic activity, influence epithelial development, and stimulate innate immunity, all key components implicated in the carcinogenic process^75^. There is a growing body of evidence regarding the role of the microbiome in PDAC, with the promotion of oncogenic signalling, genetic alterations, chronic inflammation, immunogenic TME reprogramming and secretion of microbe-derived metabolites all postulated^68^. In light of the increasing incidence of PDAC and the poor prognosis, studies defining the faecal, oral, duodenal, intratumoral, and biliary microbiota in PDAC are increasingly vital^76^. Whilst in the last two decades we have seen an explosion of interest in the microbiome and its association with PDAC as a potential resource for diagnostics, prognostication, and therapeutic strategies, it remains a daunting prospect with much yet to understand.

This systematic review has qualitatively described and summarised microbial PDAC biomarkers from organ compartments, with most compared to control subjects. Yet there are some conflicting findings within the available literature (Table 2). Taking the bile microbiome as an example, some studies have found significant differences in bile microbial signatures between PDAC and benign and/or other solid tumours of the hepatopancreatobiliary tract^41,62,63,77^ whilst other work contradict this^40^. Furthermore, there are some inconsistencies in terms of signatures identified and variability in standard annotation pipelines^41,62,63,77^ and comparing the positive findings between studies is challenging. This is in part due to a lack of publicly available raw biodata from studies coupled with the fact constituents of the bile microbiome are being described inconsistently in studies, at a class^62^, phyla^62^, or genus level^40,41,44,63^. This was the case across studies investigating compartments other than bile (Table 2). For example, in intratumoural PDAC signatures; Nalluri *et al*.^46^ described abundant families of bacteria, that is, *Ruminococcaceae, Staphylococcaceae*, *and Bacillaceae*, whereas Yu D *et al*.^78^ reported significant genera. Altogether, this inevitably limits the ability to compare study findings. A striking finding was the lack of publicly available repositories, as only 28 studies made the raw data available, with or without the accompanying metadata supporting file. The interdisciplinary and multiomics nature of the field can make resources difficult to identify and share. However, it is best practice that microbial community studies deposit at least one minimally processed and one appropriately quality-controlled sequence dataset^79^. This has now become compulsory for submission to reputable journals and highlights a need to establish consensus guidelines on extracting microbiome data and analysis. Future studies should identify more than one classification and publish their raw microbiome biodata, which will facilitate statistical analysis. This is also most certainly a limitation of this systematic review, that is, the heterogeneity of the presentation of results to taxonomic groups coupled with the lack of available datasets and the intrinsic selection bias between studies. Another consideration is that we have chosen to look at sequencing data from various compartments throughout the body. What this demonstrates is that several compartments throughout the GI tracts have clear unique microbial shifts, which could provide us with multiple avenues to explore further in terms of diagnostic and prognostic applications. However, there remains marked interindividual and intraindividual variability within cohorts across studies as well as variability in the underpinning methodology deployed to assess the microbiome. Making it ever more complicated to identify and validate relevant microbiota predictors of disease.

Furthermore, despite the growing amount of data provided by studies, the findings between studies varied considerably, irrespective of the microbiome compartment analysed (Table 2). The reasoning for this is most certainly multifaceted. Of course, this may be in part due to the small sample size of most studies but also due to the interheterogeneity and intraheterogeneity in cohorts. Naturally, given the complexity of the microbiome, small sample sizes are unlikely to account for, nor capture, the nuances associated with many clinicopathological factors and the microbiome in PDAC nuances we do not yet fully understand or appreciate. However, we are beginning to uncover some of these in PDAC. For example, Guo *et al*. have shown intratumoral transcriptome-based subtype-dependent distinct microbial communities. No other study investigating the intratumoral microbiome has provided cohort data indicating whether cohorts were basal-like, classical-like, or mixed subtypes^19,42,44,45,47,48,73^. Other examples include the implications of therapies on the PDAC microbiome. Logically, therapies have implications for the microbiome, whether that be invasive therapies like instrumentation of the CBD via ERCP/PTC, or systemic therapies like antibiotics, prebiotics or probiotics, immunotherapy, and chemotherapy. Work has shown both CBD stenting and neoadjuvant chemotherapy have impacts on the microbiome in PDAC^44,46,49^. Furthermore, the anatomical location of the tumour can also influence microbiome changes in PDAC^80^. It should also be noted that the microbiome composition in many compartments is influenced by many other environmental factors such as geographical location, diet, antibiotic therapy, and proton pump inhibitors^44,81^. Other intrinsic host factors that have been shown to have implications on the microbiome include age, sex, genetics, hormones, and bile acids^82,83^. Yet surprisingly, there is a paucity of available data relating to cohort demographic and clinicopathological variables across most studies included in this review, limiting the contextualisation of results between studies. There is also a lack of standardisation and clarity in the methodology of most studies relating to recent antimicrobial use, exposure to systemic chemotherapeutics, exposure to invasive treatments, and explicitly describing the stage of disease of cohorts. The microbiome is likely of a dynamic composition with alterations as the disease progresses and in response to therapies. If we do not capture such information in future analyses, it may compound the daunting complexity the research community is faced with in understanding the microbiome in PDAC and the interplay between environment, host, and the disease. In a bid to translate the clinical relevance of study findings, we would urge future studies to include as much clinically relevant cohort information as possible in a standardised manner. Again, this highlights the need for consensus guidelines on relevant cohort data which should be included in studies investigating the microbiome in PDAC.

16s rRNA gene sequencing method was used to assess the composition of the microbiota in the 54 papers included in this review. The exclusion criteria may have led to bias against older techniques, as studies published before 2000 were rejected. However, including a variety of methods may confound the comparison of results, due to the higher accuracy associated with modern 16srRNA sequencing and annotation techniques. 16SrRNA has been a mainstay of sequence-based bacterial analysis for two decades because of its low cost and generally reliable performance for identifying overall microbiome compositions^8^. Sequencing of 1500 base pairs (full gene length), and identification of base substitutions between copies of the 16S gene, allows taxonomic resolution to the species and strain level^9^. Despite this, the method is not ideal for detecting strains for epidemiological purposes or of specific virulence^84^. All of the studies sequenced only part of the gene, producing short sequences (≤300 bases), except one study^43^. There are clear limitations to using hypervariable regions of the 16S rRNA gene rather than full-length read sequencing technology. The full 16S gene comprises nine variable regions interspersed and provides better taxonomic resolution^9^. This would have provided a better analysis of alpha-diversity, relative abundance frequency, and identification accuracy at the species level^9,77^. Next-generation sequencing methods can exhibit the risk of detecting environmental contaminations, that otherwise significantly confound the findings of low biomass PDAC tumour samples^85–87^. A common bacteria published across multiple studies with PDAC is the phylum *Proteobacteria*. This comprises *Gammaproteobacteria* including *Enterobacteriaceae*, commensal bacteria inhabiting the intestine, and *Alphaproteobacteria* ^87^. The latter is an environmental bacterium, which is often published as tumoural microbes^86–88^. The majority of the studies within this review did not discuss the removal of contaminants within the method. This raises a major concern and pitfall of sequencing low microbial biomass samples. Eisenhofer *et al*.^86^ identified key measures that researchers can implement to reduce the impact of contaminant DNA and cross-contamination during microbiome research. The researchers designed a RIDE checklist (Report–Include–Determine–Explore), a minimum standards checklist for low microbial biomass microbiome studies^86^. The implication of negative controls is the most essential step for controlling contamination and these results should be made readily available.

The focus of studies described in this systematic review is most certainly on interrogating the compartmental microbiome in PDAC in terms of understanding its composition and utility as a biomarker for diagnostics, stratification, and prognostication. We have condensed the data available to draw practical conclusions, for clinical purposes. It is crucial to emphasise the need for standardising these experiments moving forward within our community. While these sequencing experiments are descriptive of the microbial composition in pancreatic cancer, they do not provide clarity on the underlying mechanisms whereby the microbiome might influence the TME and disease. What is of great interest is establishing a causal relationship between PDAC and the microbiome, with obvious applications. Several key studies, have begun to shed light on causality and the influence the microbiome constituents and their produced metabolites could play in tumorigenesis and influencing outcomes^89–103^.

It has been demonstrated that microbial/bacterial produced metabolites drive the immune landscape of the PDAC TME and can potentially influence PDAC outcomes^89^. For example, the transcriptional regulator, Aryl hydrocarbon receptor (AhR), is a modulator of immunity and AhR expression is elevated in myeloid-lineage cells relative to other cell types^90,91^. In particular, AhR activation can drive macrophages to acquire an immunosuppressive phenotype through the upregulation of immune-suppressive cytokines interleukin (IL)-10^91^ and IL-11^92^, as well as stimulating the expression of arginase (Arg1), transforming growth factor (TGF)-α, and TGF-β^93,94^. Indole (and its related compounds) are an immunologically important class of bacterial-metabolites derived by the metabolisation of amino acid tryptophan (Trp) to indole/related compounds^91,104^, which ultimately activates AhR^105^. Hezaveh *et al*.^89^ have demonstrated in a PDAC murine model, that macrophage AhR deletion *(Lyz2* ^*cre/+*^ *Ahr* ^*fl/fl*^ mice) results in proinflammatory polarization of TAMs, expression of PD-L1 in TAMS, and activated CD8+ T cell infiltration in the PDAC TME, ultimately resulting in reduced tumour burden compared to controls. Most importantly, this study demonstrated that the increased alpha-diversity and reduced abundance of the genus *Lactobacillus* (in particular indole producing *Lactobacillus murinus* species) in the gut microbiome of antimicrobial-treated PDAC mice models, phenocopied observations in *Lyz2* ^*cre/+*^ *Ahr* ^*fl/fl*^ mice, compared to controls. Whilst this study was not able to formally investigate the intratumoral or other compartmental microbiome profiles associated with antimicrobial administration beyond the gut in mice models, it does suggest a potential link between the antimicrobial modulation of the lactobacillus genus in the gut microbiome and intratumoral immunity. Indeed, it has been shown that some Lactobacillus species like *L. murinus* and *L. reuteri* can produce anti-inflammatory metabolites like indoles^89,95,96^. Furthermore, Hezaveg *et al*. transplantated these indole-producing bacteria in a germ-free PDAC mice model in order to showcase that they can promote tumour growth through an immunosuppressive programme in PDAC-TAMs. In this model, the TAMs demonstrated increased AhR activity, and expression of protumour genes Arg1, Ido1, and IL-10, whilst the TME had an overall decrease in intratumoral CD8+ T cells, TNF-a production and an increase in Myeloid-derived suppressor cells (MDSCs), compared to controls. Indeed, indole-producing lactobacillus species may influence immunity in the TME.

*L. reuteri* metabolites are also involved with distinct energy metabolism pathways, which have previously been implicated in PDAC. For example, Mendez *et al*.^102^ performed a metabolomic study on the analysis of the microbiome in a genetic KPC mouse model for PDAC. Faecal samples were collected at 2, 3, and 4 months of age and a combination of 16s rRNA pyrosequencing and whole-genome sequencing of gut faecal microbiota was performed. The authors identified bacterial species and primary microbial metabolites that harbour distinct energy metabolism pathways. At ages 2–4 months KPC mice experienced disruptions in metabolic processes related to the production of polyamines and pyrimidines that are considered a marker for neoplastic progression^102^. Serum polyamine levels were also significantly elevated in PDAC patients. The study concluded that there is a shift in the microbial composition early in the tumour development timeline (histological confirmation of PanINs and dysplasia). In addition, specific bacteria such as *Lactobacillus reuteri* can control the tumorigenesis by managing polyamine metabolism and the release of pyrimidines^102^.

Several other bacterial taxa from the Bacteroides, Bifidobacterium, and Clostridium genera are producers of indoles^97,98,106^. Studies included in this systematic review have identified many of these genera as enriched in PDAC tissue^49^, faecal samples^51^, and duodenal^11,36^ and oral samples^30^. In particular, one study has shown that a higher abundance of particular Clostridium species in the PDAC tissue is associated with decreased overall survival^38^. Whilst the molecular mechanisms underpinning these findings were not investigated, this finding could be contextualised and at least, in part, explained by the influence that indole-producing bacteria have on the TME and tumour growth. This hypothesis is further strengthened by work demonstrating an increased relative abundance of indole-producing bacteria or genera (e.g. Lactobacillus, Bifidobacterum genera, Bacteroides coprophilus, and Faecalibacterium prausnitzi) in STS versus long term survivors of resected PDAC^20,89^. Whether indoles and the AcR pathway in PDAC is of significance in humans remains to be determined, especially if we consider the fact there is a clear difference in AhR Ligand binding affinities between mice and humans^107,108^. Another gut-microbe-derived metabolite trimethylamine N-Oxide (TMAO) has been implicated in PDAC^109^. TMAO is a consequence of dietary choline being converted to trimethylamine (TMA) by the gut bacterial enzyme, choline TMAlyase. TMA is oxidised in the liver, via the portal circulation to TMAO^110^. This process is entirely dependent on intestinal bacteria^111^. Employing an orthotopic mouse model of PDAC, one study has demonstrated that TMAO relieves immunosuppression in the PDAC TME^109^. TMAO and TMA were observed to be a direct driver of an immunostimulatory phenotype in TAMs, as well as other myeloid cells like MDSCs and dendritic cells (DCs), ultimately driving the cancer-killing effector T-cell response in the TME. Mechanistically, the immunostimulation of the TME was dependent on the type-I interferon (IFN) pathway. It is known that Type-I IFN activation restricts TAMs whilst promoting polarisation towards an immunostimulatory phenotype. Furthermore, the delivery of TMAO intraperitoneally or via dietary choline supplements or delivery of the microbial TMA-Lyase, to orthotopic PDAC-bearing mice, resulted in reduced tumour growth and improved overall survival.

Thus, this metabolite derived from gut bacteria may suppress tumour growth via a reconfiguration of the tumour milieu to an immune-activated state. This study also demonstrated that the antitumour effect of TMAO observed in PDAC is dependent on the bacterial enzyme CutC. Several bacterial taxa belonging to Clostridia, Bacilli, Desulfovibrionia, and Gammaproteobacteria contain the CutC gene and are significant producers of TMA and contribute to circulating TMAO levels^112–114^. Mirji *et al*. used a gene-targeted assay for choline TMAlyase (CutC) in the tumour microbiome 16S ribosomal RNA gene sequencing data of the intratumoral microbiome in PDAC LTS versus STS, from a study reported in this review^20,109^. At a genus level, they observed a greater relative abundance of *Bacillus* and *Paenibacillus* in LTS suggesting the presence of such TMA-producing bacteria correlates with improved survival in PDAC.

Microbiota-derived indoles have also been implicated in chemotherapy response. For example, Tintelnot *et al*.^99^ has identified another microbiota-derived metabolite named indole-3-acetic acid (3-IAA) as a key amplifier of the response to chemotherapy in PDAC. The intestinal microbiota of 23 patients with metastatic PDAC was sequenced before the start of chemotherapy. The microbiome of patients who showed a response to chemotherapy treatment (R=10) differed from those who did not respond (NR=12), and this was associated with improved survival. To study a potential cause-effect relationship between the microbiota and chemotherapy response, faecal microbiota transplantation from the first ten recruited R (responders) and NR (nonresponders) patients was performed into gnotobiotic mice, followed by orthotopic injection of Pdx1-Cre, LSL-KRASG12D, LSL-Trp53R172H/+ (KPC) pancreatic cancer cells^99^. The researchers found tumour development in mice that had only been exposed to the microbiota of NR patients with (5-FU, irinotecan and oxaliplatin; FIRINOX)^99^. A targeted metabolomic screen using liquid chromatography coupled to mass spectrometry was performed in the matched plasma serum of these patients. Screening identified the tryptophan metabolite 3-IAA as one of the most abundant in the serum of R patients and R microbiota-colonised gnotobiotic mice^99^. This study suggests that *B. fragilis* and *B. thetaiotaomicron* are enriched in the microbiota of treatment responders and were able to produce 3-IAA^99^. The authors discovered that high concentrations of 3-IAA resulted in a reduction, in the proliferation of PDAC cells due to the process by which Neutrophils release myeloperoxidase (MPO) causing cellular damage, through the oxidation of biomolecules. Firstly, bacteria within the gut produce 3-IAA from food-derived tryptophan (Trp)^99^. 3-IAA translocates to the tumour site by circulation and undergoes oxidation into toxic molecules (3-IAAP) by MPO and cytotoxic anticancer drugs (FOLFIRINOX) within intratumoral neutrophils. Reactive oxygen species (ROS) start to accumulate as a result of reducing the levels of enzymes that break down ROS, such, as glutathione peroxidase 3 and glutathione peroxidase 7 (GPX3 and GPX7). Tintelnot *et al*.^99^ demonstrated that accumulation of ROS in pancreatic tumour cells was central for the therapeutic efficacy of FOLFIRINOX. As the ROS levels increased, this suppressed the autophagy pathway that directly reduced the proliferation of tumour cells. In summary, the authors identified a specific microbiota-derived metabolite and MPO present during chemotherapy treatment that led to the accumulation of ROS and directly stopped the proliferation of tumour cells^99^. Future clinical trials that aim to increase 3-IAA or MPO during chemotherapy may lead to an improvement in OS in PDAC. Similar studies such as Yao *et al*.^100^ found that a gut microbial metabolite named butyrate promoted CD8+ T cell immunity by activating the IL-12 signalling pathway. Han *et al*.^101^ performed in vivo studies which showed that *Lactobacillus reuteri* administration reduced colorectal tumourigenesis by releasing a tryptophan catabolite, indole-3-lactic acid (ILA). This microbial metabolite inhibits T helper 17 cell differentiation by downregulating the IL-17 signalling pathway.

Li *et al*.^103^ explored the spatial relationship between microbes, tumour, and immune cells and identified a distinct microbial- T cell interaction that influences tumourigenesis. The authors performed multiple imaging studies of the TME in human PDAC to identify the distribution of microbes and the immune cells. In addition to RNA sequencing that identified molecular signatures in tumour nests that contained microbes in PDAC, the spatial dependency between the microbes and immune cells within the TME were examined in mouse models of T cell-poor and T cell-enriched tumours^103^. Furthermore, the expression of LPS (lipopolysaccharide) for gram-negative bacteria was found in association with CD8+ T cell infiltration and mostly limited to the stromal microenvironment in the PDAC TME^103^, highlighting that T cells may support microbial presence in tumours by actively transporting bacteria and by recruiting other cells that harbour intracellular bacteria^103^. The authors went on to characterize PDAC intratumoural communities as CD8+ T cell enriched (hot) or poor (cold) depending on immune cells infiltration and CK19+ cancer cells expression of Ki-67. RNA sequencing from the hot and cold tumour nests, and principal-component analysis of differentially expressed genes (DEGs) showed that cold and hot stroma clustered distinctly. Bacterial 16S rRNA showed that hot tumour nests had greater abundance of bacteria and these findings were consistent with the increased LPS localization to CD8+ T cell-enriched tumour regions. The hot stroma region showed an enrichment of genes associated with response to bacterium (GO: 0009617) and genes involved in T and B cell chemotaxis and immune regulation. In summary these results showed that the variability, in gene expression within PDAC is influenced by spatial factors and correlates with the presence of microbes and CD8+ T cells in tumour regions^103^. In addition, hot tumour epithelium sites exhibited increased levels of PIGR and CD74 receptors, which are known for triggering an immune response, against microbes^103^. The causal relationship between intra-tumoural T cells and microbes were investigated using PDAC cell lines which were injected orthotopically into mice. The authors demonstrated that specific microbial communities were found in T cell-poor and enriched tumours. For instance, microbes in T cell-poor tumours supported tumour growth, whereas microbes in T cell-enriched tumours facilitated B cell infiltration and the upregulation of intratumoral macrophages^103^. Removing T cells and disrupting the immunological environment of the tumour directly led to a reduction of intra-tumoural bacteria, suggesting that there is a spatial coupling between intratumoural bacteria and immune cells within PDAC tumours and that T cells are necessary for the accumulation of intratumoral microbes.

## Conclusion

The microbiome may provide numerous novel therapeutic targets for the treatment of PDAC; however, this research area is still in its infancy. After a review of the selected articles and consideration of the limitations described, it is not possible to state a universal 16S rRNA gene microbial signature that can be used for PDAC screening. However, the microbiome has the potential to become a prognostic biomarker in the future. The challenge in this field is to shape the available microbial data into targetable signatures. Making sequenced data readily available is critical, allowing the potential for meta-analysis of 16S rRNA gene and metagenomic PDAC studies. This needs to be coupled with the coordinated standardisation of data collected relating to demographics and clinicopathological factors associated with PDAC as well as other environmental and host factors. In doing so, results observed in studies can be contextualised into meaningful findings with clinical relevance. The identification of signatures may further the aim of ultimately, designing interventional microbiome PDAC studies. Like many other human diseases currently studied, it is clear that in the future the microbiome in PDAC has potential for identifying disease associations and developing therapeutic treatments.

## Ethical approval and consent

Not applicable to this systematic review study.

## Consent

Not applicable to this systematic review study.

## Source of funding

This research was funded by the Royal College of Surgeons of England the Marx Family Trust Research Fellowship and Mason Medical Research Trust grant number RC3597.

## Author contribution

N.M.: conceptualization, investigation, data curation, methodology, formal analysis, and writing; T.C. and C.S.: joint second authors: investigation, data curation, and writing; J.H.-S., J.O’B., and M.-D.J.: investigation and data curation; B.W.: writing – review and editing; S.S.: supervised study design; J.I.J., K.J.R., E.V., S.S., E.G., N.E.A., and T.A.R.: supervised study design, and critically corrected the manuscript; A.D., V.P., A.E.F., and D.D.: supervised study design, data analysis, and critically corrected the manuscript.

## Conflicts of interest disclosure

The authors declare no conflict of interest.

## Research registration unique identifying number (UIN)

Prospero UIN: CRD42024498780.

## Guarantor

Professor Adam E. Frampton BSc (Hons) MSc PhD MBBS MRCSEd FRCS (Gen Surg) FRSB DipMedTox, Associate Professor in Surgical Oncology and Head of Oncology, Department of Clinical and Experimental Medicine, University of Surrey, Guildford, Surrey, UK. E-mail: adam.frampton@surrey.ac.uk

## Data availability statement

The data that support the findings of this study are available from the corresponding author upon reasonable request.

## Provenance and peer review

Not commissioned, externally peer-reviewed.

## Supplementary Material

SUPPLEMENTARY MATERIAL
