# Supplementary material for: The microbial composition of pancreatic ductal adenocarcinoma: a systematic review of 16S rRNA gene sequencing
Source: Int J Surg. 2024 Jun 14;110(10):6771–99. doi: 10.1097/JS9.0000000000001762 (PMC11487005; doi:10.1097/JS9.0000000000001762)
Supplement: SUPPLEMENTARY MATERIAL [file js9-110-6771-s002.docx]

**Identification of studies via databases and registers**

Records removed *before screening*:

Duplicate records removed (n = 702)

Records identified from*:

Databases (n = 1727)

**Identification**

Records excluded**

(n = 880)

Records screened

(n = 1025)

Reports not retrieved

(n = 7)

Reports sought for retrieval

(n = 145)

**Screening**

Reports excluded:

Wrong study design 1 (n = 79)

Wrong population 2 (n = 4)

Wrong outcome 3 (n = 12)

Removal of Duplicates 4 (n=5)

Reports assessed for eligibility

(n = 138)

Studies included in review

(n = 38)

Reports of included studies

(n = 38)

**Included**

*Consider, if feasible to do so, reporting the number of records identified from each database or register searched (rather than the total number across all databases/registers).

**If automation tools were used, indicate how many records were excluded by a human and how many were excluded by automation tools.

*From:*  Page MJ, McKenzie JE, Bossuyt PM, Boutron I, Hoffmann TC, Mulrow CD, et al. The PRISMA 2020 statement: an updated guideline for reporting systematic reviews. BMJ 2021;372:n71. doi: 10.1136/bmj.n71

For more information, visit: <http://www.prisma-statement.org/>
